# Comparative Gene Expression Analysis of Malignant Mesothelioma and Lung Adenocarcinomas Induced by Multi-Walled Carbon Nanotube-7 and Double-Walled Carbon Nanotubes in Rats: Distinct Molecular Signatures and Canonical Pathways

**DOI:** 10.3390/nano15231806

**Published:** 2025-11-29

**Authors:** Min Gi, Shugo Suzuki, Dina Mourad Saleh, Omnia Hosny Mohamed Ahmed, William T. Alexander, Masaki Fujioka, Arpamas Vachiraarunwong, Runjie Guo, Guiyu Qiu, Ikue Noura, Anna Kakehashi, Xiao-Li Xie, Shuji Tsuruoka, Akihiko Hirose, Aya Naiki-Ito, Hiroyuki Tsuda, Hideki Wanibuchi

**Affiliations:** 1 Department of Environmental Risk Assessment, Graduate School of Medicine, Osaka Metropolitan University, Osaka 545-8585, Japan; m22438g@omu.ac.jp (A.V.); sy23105k@st.omu.ac.jp (R.G.); si22394d@st.omu.ac.jp (G.Q.); wani@omu.ac.jp (H.W.); 2 Department of Molecular Pathology, Graduate School of Medicine, Osaka Metropolitan University, Osaka 545-8585, Japan; f21049w@omu.ac.jp (S.S.); b21405o@omu.ac.jp (M.F.); sx23713o@st.omu.ac.jp (I.N.); anna-k@omu.ac.jp (A.K.); 3 Nanotoxicology Project, Nagoya City University Graduate School of Medical Sciences, Nagoya 467-8603, Japan; drdina@aun.edu.eg (D.M.S.); omnia.hosny@aswu.edu.eg (O.H.M.A.); william@phar.nagoya-cu.ac.jp (W.T.A.); htsuda@phar.nagoya-cu.ac.jp (H.T.); 4 Department of Forensic Medicine and Clinical Toxicology, Faculty of Medicine, Assiut University, Assiut 71515, Egypt; 5 Center of Excellence for Toxicological Testing, Mammalian and Aquatic Toxicology Department, Central Agricultural Pesticides Lab, Agricultural Research Center, Dokki, Giza 12618, Egypt; 6 Department of Forensic Medicine and Clinical Toxicology, Faculty of Medicine, Aswan University, Aswan 81528, Egypt; 7 Department of Experimental Pathology and Tumor Biology, Graduate School of Medical Sciences, Nagoya City University, Nagoya 467-8601, Japan; ayaito@med.nagoya-cu.ac.jp; 8 Department of Toxicology, School of Public Health, Southern Medical University, Guangzhou 510515, China; xiexiaoli1999@126.com; 9 Neura Inc., Tokyo 100-0005, Japan; suruoka.saitama@gmail.com; 10 Chemicals Assessment and Research Center, Chemicals Evaluation and Research Institute, Tokyo 112-0004, Japan; akihikoh@dranihs.net

**Keywords:** 1.5 µm DWCNT, 7 µm DWCNT, MWCNT-7, lung adenocarcinoma, malignant mesothelioma, gene expression profiling

## Abstract

Although numerous experimental studies have demonstrated the carcinogenic potential of multi-walled carbon nanotubes (MWCNTs) in lungs, the underlying molecular mechanisms—especially gene expression changes associated with different tumor types—remain poorly characterized. To elucidate the molecular signatures associated with MWCNT-induced carcinogenesis, we performed microarray-based gene expression profiling of rat lung tumors induced by MWCNT-7, including both adenocarcinoma (ADC) and malignant mesothelioma (MM), as well as ADCs induced by two types of double-walled CNTs (DWCNTs) differing in fiber length (1.5 µm and 7 µm). Hierarchical clustering revealed that the MWCNT-7-induced MM exhibited a gene expression profile distinct from the ADCs. The ADCs induced by the DWCNTs and the ADC induced by MWCNT-7 shared several pathways that were distinct from those of the MWCNT-7 induced MM. The distinct pathways upregulated in the ADCs versus the MM support the conclusion that MWCNT-induced ADCs arise through distinct biological mechanisms compared to MWCNT-induced MMs and identified tumor-type-specific biomarker candidates: complement factor I (CFI) and secreted phosphoprotein 1 (SPP1) for ADCs, and fibronectin 1 (FN1) for MM. In addition, the gene expression profiles of the ADCs induced by the three fiber types indicate that both types of thin flexible DWCNTs used in the present study promoted a number of carcinogenic pathways in the rat lung that were also promoted by MWCNT-7, which is a class 2B carcinogen. These results support the conclusion that DWCNTs are carcinogenic in the rat lung and highlight the importance of further assessments of the potential lung carcinogenicity of inhaled thin flexible CNTs.

## 1. Introduction

Carbon nanotubes (CNTs) are cylindrical nanomaterials composed of carbon atoms arranged in a hexagonal lattice. CNTs are broadly classified into single-walled carbon nanotubes (SWCNTs) and multi-walled carbon nanotubes (MWCNTs) based on the number of concentric graphene layers. Their unique structural configuration imparts exceptional mechanical strength, thermal conductivity, and electrical properties, which have led to their widespread application in various fields, including electronics, materials science, energy storage, and biomedicine [1]. As industrial demand has increased, large quantities of CNTs are now being manufactured globally [2]. Notably, the structural and biological similarities between certain MWCNTs—particularly multi-walled carbon nanotube-7 (MWCNT-7)—and asbestos, a known human carcinogen, have raised significant concerns about potential human health risks, particularly in occupational settings where inhalation exposure may occur [3,4]. However, only a few long-term studies have been carried out, investigating the carcinogenic potential of inhaled CNTs [5,6].

Among the various CNTs, MWCNT-7 has been the most extensively studied and is classified by the International Agency for Research on Cancer as a Group 2B carcinogen (possibly carcinogenic to humans) based on sufficient evidence of carcinogenicity in experimental animals [7,8]: MWCNT-7 induced malignant mesothelioma following intraperitoneal injection in p53+/− mice [9,10], intraperitoneal injection in rats (referred to as NT50a in the study; see Table 3.2 in IARC volume 111 [7]), [11], and intrascrotal injection in rats [12], and exposure to MWCNT-7 by whole-body inhalation for 15 days promoted lung carcinogenesis initiated with methylcholanthrene [13]. A later inhalation study demonstrated that 2 years of whole-body inhalation exposure to MWCNT-7 induced lung adenocarcinomas (ADCs) in rats [14], confirming the classification of MWCNT-7 by IARC as a Group 2B carcinogen.

The carcinogenic potential of various MWCNTs has been comprehensively reviewed [5]. To date, only a single long-term carcinogenicity study of CNTs using whole-body inhalation exposure has been reported [14]. Another method of administration of test material to the lung is intratracheal instillation (also referred to as intratracheal intrapulmonary spraying or TIPS). The TIPS method is a practical and well-established alternative to inhalation studies for evaluating the pulmonary toxicity of nanomaterials [15,16,17,18,19]. An initial study demonstrated that the administration of MWCNT-7 into the lung can induce malignant mesothelioma (MM) in rats [15], and a later study administering MWCNT-7 via intratracheal instillation once every 4 weeks over the course of 2 years demonstrated that MWCNT-7 can induce both lung ADCs and MMs [20]. Using TIPS, another MWCNT, MWCNT-N, which is structurally similar to MWCNT-7, was also shown to induce both lung ADCs and MMs in rats [21].

Studies administering CNTs via TIPS have also indicated that thinner flexible MWCNTs possess tumorigenic potential in the rat lung: See Table 5 in Ahmed et al., 2025 [5]. However, while thick, rigid MWCNT-7 and MWCNT-N can induce both lung ADCs and MMs, the thin flexible CNTs are associated only with the development of lung ADCs. Currently, only three long-term studies examining the effects of lung exposure to thin flexible CNTs have been carried out: one study examined the effects of MWCNT-B [16], and two studies examined the effects of DWCNTs [6,17].

An important result of the recent two-year carcinogenicity study of DWCNTs of different lengths is that the carcinogenicity of the DWCNTs was inversely proportional to the total dose of fibers administered [6]. In that study, rats were administered the same number of 1.5 µm fibers, 7 µm fibers, and 15 µm fibers. Consequently, the total dose of fibers administered was 50.4 µg of the 1.5 µm DWCNT, 232.3 µg of the 7 µm DWCNT, and 504.0 µg of the 15 mm DWCNT, and the number of rats that developed lung tumors was 4/12 rats in the 1.5 µm DWCNT group, 3/8 rats in the 7 µm DWCNT group, and 2/10 rats in the 15 µm DWCNT group. This corresponds to 6.6 carcinomas per rat per mg of the 1.5 µm DWCNT, 1.6 carcinomas per rat per mg of the 7 µm DWCNT, and 0.4 carcinomas per rat per mg of the 15 µm DWCNT. Thus, it was important to investigate whether these tumors arose spontaneously or were induced by the administration of DWCNT into the lung.

In addition, in the above long-term carcinogenicity study, 11 of 15 rats administered with MWCNT-7 died from MM, and the remaining 4 rats were sacrificed at week 75. One of these four rats had developed MM, and two rats developed ADC. This allowed us to examine the genetic profile of these two tumor types induced by the same CNT fiber, MWCNT-7. This also allows us to compare the genetic profiles of DWCNT-induced tumors with the MWCNT-7-induced tumors.

Although persistent cytotoxicity, inflammation, fibrosis, and reactive oxygen species generation have been implicated in MWCNT-induced lung carcinogenesis [22,23,24], the underlying molecular mechanisms, particularly the gene expression changes associated with different tumor types, remain poorly characterized. Elucidating these molecular pathways will facilitate improving MWCNT-related risk assessments and identifying the molecular biomarkers of MWCNT-induced lung cancer.

In the present study, we performed the microarray-based gene expression profiling of a lung ADC and a pleural MM induced by MWCNT-7 and lung ADCs induced by the 1.5 µm and 7 µm DWCNTs: Lung tumors from the 15 µm DWCNT-administered rats were not available for RNA analysis. Our objective was to characterize the molecular signatures associated with MWCNT-7-induced lung and mesothelial carcinogenesis and to gain insights into the carcinogenic potential of structurally diverse thick, rigid, and thin flexible MWCNTs.

## 2. Materials and Methods

### 2.1. MWCNTs

Three types of MWCNTs were used in this study. Two types of DWCNTs were obtained from Neura Inc., Tokyo, Japan: 1.5 µm DWCNT (1.5 μm length) and 7 µm DWCNT (7 μm length). According to the supplier, both DWCNTs contained iron levels below the detectable limit. MWCNT-7 (more than 40 layers, 6.5 ± 2.4 μm length) was supplied by Mitsui Chemicals Inc. (Tokyo, Japan) with an iron content of 0.3% by weight [17]. A description of the agglomerates formed by 1.5 µm DWCNT, 7 µm DWCNT, and MWCNT-7 is given in Ahmed et al., 2025 [6].

### 2.2. Animals

Nine-week-old male F344 rats were purchased from Charles River Japan Inc. (Yokohama, Japan). The animals were housed in the Center for Experimental Animal Science of Nagoya City University Medical School, maintained according to a 12 h light–dark cycle, and received Oriental MF basal diet (Oriental Yeast Co., Tokyo, Japan) and tap water ad libitum. The experimental protocol was approved by the Animal Care and Use Committee of Nagoya City University Graduate School of Medical Sciences (protocol code 22-014 and data of approval 21 September 2022), and research was conducted according to the Guidelines for the Care and Use of Laboratory Animals of Nagoya City University. The experiment was started after a 1-week acclimation and quarantine period.

### 2.3. Preparation of the CNT Suspension

CNT suspensions were prepared as described previously [17]. Briefly, CNTs (1.5 µm DWCNT, 7 µm DWCNT, and MWCNT-7) were weighed and dispersed in tert-butyl alcohol. Shortly before administration, the tert-butyl alcohol was removed, and the CNTs were suspended in saline containing 0.5% Pluronic F-68 (Sigma-Aldrich, St. Louis, MO, USA). Immediately prior to administration, the suspensions were sonicated for 30 min using a Tomy Ultrasonic disruptor, UD-211, equipped with a TP-040 micro-tip (Tomy Seiko Co., Ltd., Tokyo, Japan) at a power setting of 4, to ensure stable dispersion of the CNT fibers.

### 2.4. Experimental Design

In the 104-week study [6], 16-20 rats were randomly assigned to each of the following four groups: vehicle control, saline with 0.5% Pluronic F-68 (18 rats), 1.5 µm DWCNT (18 rats), 7 µm DWCNT (16 rats), and MWCNT-7 (20 rats). The lower number of rats in the 1.5 µm DWCNT and 7 µm DWCNT groups was due to the low availability of these DWCNTs, which were specifically produced by Neura Inc., Tokyo, Japan, for the 104-week study. Rats were administered the CNT solutions by TIPS as previously described [17]. Briefly, rats were anesthetized with 3% isoflurane and administered 0.5 mL vehicle or CNT suspensions using a micro-sprayer (series IA–1B Intratracheal Aerosolizer Penn-century, Philadelphia, PA, USA). Representative transmission electron microscopy images of each CNT type used in this study are available in our companion long-term carcinogenicity manuscript [6]. The doses per administration were 6.3 µg/2.660 × 10^12^ 1.5 µm DWCNT fibers per rat, 29.0 µg/2.720 × 10^12^ 7 µm DWCNT per rat, and 63.0 µg MWCNT-7 per rat. Rats were administered the test materials once every other day over a 15-day period, totaling eight administrations per rat. The total doses were 50.4 μg of the 1.5 µm DWCNT, 232 μg of the 7 µm DWCNT, and 504 μg of MWCNT-7. Five rats per group were sacrificed at week 6. One rat in the vehicle group and three rats in the 7 µm DWCNT group died during weeks 29–30 due to mechanical failure of the drinking water supply system and were removed from the study. In total, 11 rats in the MWCNT-7 group died before week 75 due to MM, and 1 rat in the 1.5 µm DWCNT group died before week 75, leaving 12 rats in the vehicle group, 12 rats in the 1.5 µm DWCNT group, 8 rats in the 7 µm DWCNT group, and 4 rats in the MWCNT group available for tumor analysis. The 4 rats in the MWCNT-7 group were sacrificed at week 75, and the rats in the vehicle and DWCNT groups were sacrificed at week 104. At the final sacrifice, all animals underwent complete necropsy and histopathological examination to evaluate long-term pulmonary effects, including tumor development. In the MWCNT-7 group, 1 developed MM and 2 rats developed ADC. In the 1.5 µm DWCNT group, 4 rats developed ADC, and no rats developed MM. In the 7 µm DWCNT group, 3 rats developed ADC, and no rats developed MM. None of the rats in the control group developed tumors. For gene expression analysis, three adenocarcinomas (ADCs) and one malignant mesothelioma (MM) with diameters greater than 3 mm that were sufficient for RNA extraction were snap-frozen in liquid nitrogen and stored at −80 °C. These included one ADC, each induced by 1.5 µm DWCNT (1.5 µm DWCNT-ADC), 7 µm DWCNT (7 µm DWCNT-ADC), and MWCNT-7 (MWCNT-7-ADC), and one MM induced by MWCNT-7 (MWCNT-7-MM). As controls, lung tissues from three individual rats in the vehicle group were also collected. Total RNA was extracted from tumor and control tissues using the RNeasy Mini Kit (QIAGEN, Hilden, Germany) according to the manufacturer’s instructions. The quality of total RNA was assessed by Cell Innovator Inc. (Fukuoka, Japan) using an Agilent 2200 TapeStation (Agilent Technologies, Santa Clara, CA, USA), and all samples had RIN values greater than 9.

### 2.5. Microarray Gene Expression Analysis

Microarray gene expression was performed using the GeneChip^®^ Rat Genome 230 2.0 Array (Affymetrix, Santa Clara, CA, USA) by Cell Innovator Inc. (Fukuoka, Japan). Raw data were processed using the Affymetrix Expression Console 1.1 software. Signal intensity values were normalized using the SST-RMA method combined with the quantile normalization algorithm. Low-intensity signals with fluorescence values below 100 were excluded during the data-cleansing step. Differentially expressed genes (DEGs) were defined as those showing a z-score ≥ 2 and a fold change ≥ 2 for upregulation or a z-score ≤ −2 and a fold change ≤ 0.5 for downregulation. Significant associations between DEGs and canonical pathways were determined using a right-tailed Fisher’s exact test, with significance at *p* < 0.05. Pathway activation or inhibition was predicted by Ingenuity Pathway Analysis (IPA), Version 01-23-01 (QIAGEN Inc., Redwood City, CA, USA) using IPA’s z-score algorithm, where a z-score ≥ 2 indicates significant activation and a z-score ≤ −2 indicates significant inhibition [25].

Biomarker candidates were identified using the Biomarker analysis in IPA, based on the Ingenuity Knowledge Base (IKB), with the following criteria: species restricted to human; tissues limited to lung; biofluids limited to bronchoalveolar lavage fluid or plasma/serum; disease context set to cancer; and biomarker applications restricted to diagnosis for the following conditions—lung adenocarcinoma, lung cancer, lung carcinoma, lung neoplasm, mesothelial neoplasm, or mesothelioma.

## 3. Results

### 3.1. DEGs in Adenocarcinoma (ADC) and Malignant Mesothelioma (MM) Induced by Different Types of MWCNTs

**Figure 1:**
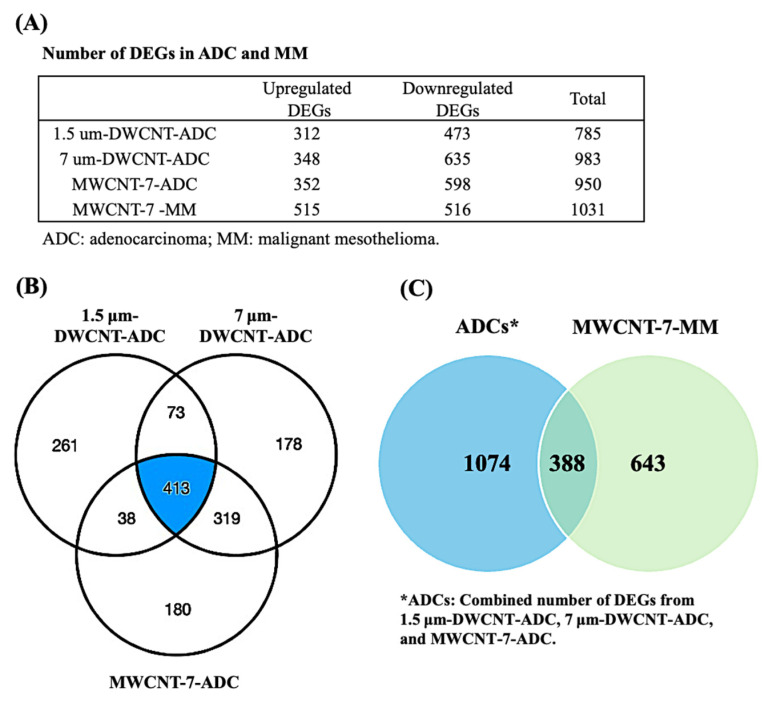
(**A**) Number of differentially expressed genes (DEGs) in adenocarcinoma (ADC) and malignant mesothelioma (MM) induced by different types of MWCNTs. (**B**) Overlap of DEGs among the three MWCNT-induced ADCs (1.5 μm DWCNT-ADC, 7 μm DWCNT-ADC, and MWCNT-7-ADC). (**C**) Overlap between the combined DEGs from the three ADCs and those from MWCNT-7-MM.

The number of DEGs identified in three ADCs and one MM induced by different types of MWCNTs is summarized in Figure 1A. In the three ADCs, 785 DEGs were identified in 1.5 μm DWCNT-ADC (312 upregulated and 473 downregulated), 983 DEGs in 7 μm DWCNT-ADC (348 upregulated and 635 downregulated), and 950 DEGs in the MWCNT-7-ADC (352 upregulated and 598 downregulated). A total of 413 DEGs were commonly shared across all three ADCs (Figure 1B), including 81 genes consistently upregulated, 330 consistently downregulated, and 2 genes (LRRN4 and S100A9) downregulated in 7 µm DWCNT-ADC and MWCNT-7-ADC, but they were upregulated in the 1.5 µm DWCNT-ADC (Table S1). Each ADC also exhibited a substantial number of unique DEGs. The ADC results indicate both shared and distinct gene expression changes among ADCs induced by structurally different MWCNTs, suggesting that fiber length and structure influence the molecular features of CNT-induced ADCs.

In the MM tumor, MWCNT-7-MM exhibited 1031 DEGs, consisting of 515 upregulated and 516 downregulated genes (Figure 1A). While 388 DEGs were commonly shared between the MM and the three ADCs, 643 DEGs (432 upregulated and 211 downregulated) were uniquely identified in the MM tumor and were not differentially expressed in any of the ADCs (Figure 1C) (Table S2). Conversely, of the 1462 DEGs representing the combined number of DEGs across the three ADC tumors, 1074 DEGs were specific to the ADCs (Figure 1C). Notably, none of the 413 DEGs commonly shared across all three ADCs were differentially expressed in the MM, indicating that these genes are specific to ADCs. This distinct distribution highlights substantial differences in gene expression profiles between MM and ADC, suggesting that CNT-induced MM and ADC may arise through divergent molecular mechanisms. These findings prompted us to further compare the canonical pathways enriched in ADCs and MM, as described in the following sections.

### 3.2. Hierarchical Clustering of Canonical Pathways Enriched in Adenocarcinomas (ADCs) and Malignant Mesothelioma (MM)

**Figure 2:**
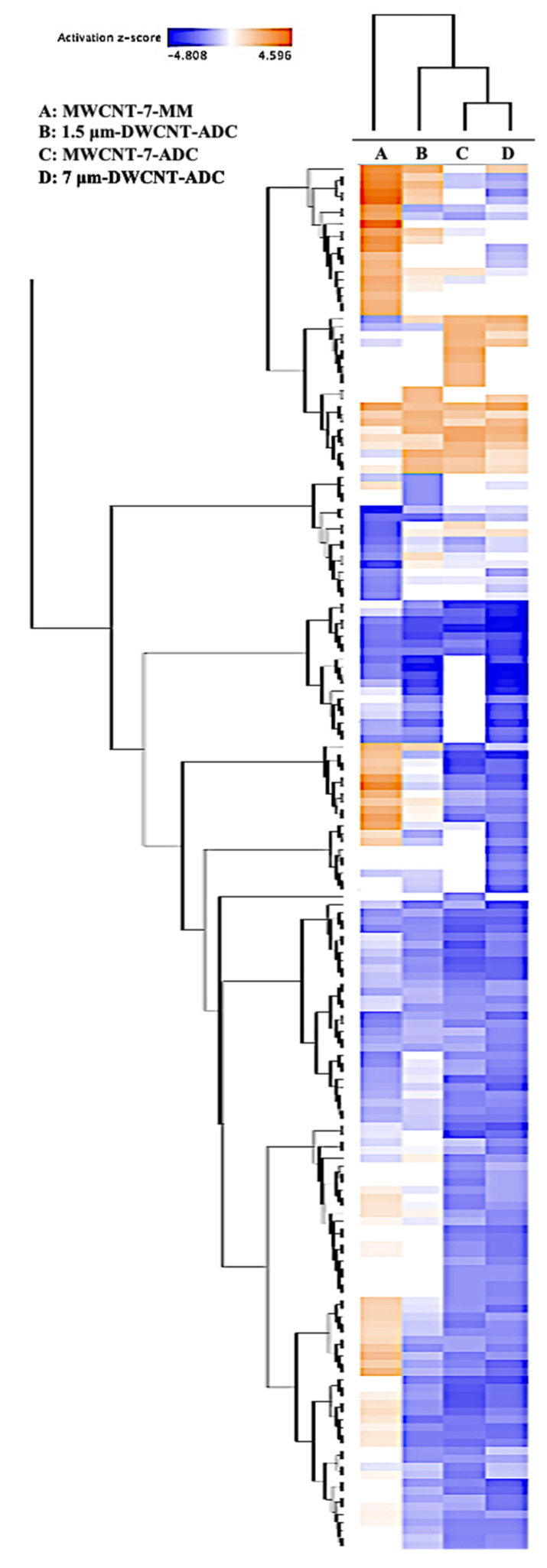
Hierarchical clustering of canonical pathways enriched in MWCNT-induced adenocarcinomas (ADC) and malignant mesothelioma (MM).

The hierarchical clustering of canonical pathways enriched in the three ADCs (1.5 μm DWCNT-ADC, 7 μm DWCNT-ADC, and MWCNT-7-ADC) and the MWCNT-7-MM revealed two distinct molecular clusters (Figure 2 and Figure S1). The first major cluster consisted exclusively of MWCNT-7-MM, indicating a pathway profile distinct from those of the ADCs. The second major cluster included all three ADCs, which exhibited more uniform and distinct pathway activation patterns. Within this cluster, 7 μm DWCNT-ADC and MWCNT-7-ADC were grouped into the same subcluster, suggesting a high degree of similarity in their enriched pathways. In contrast, 1.5 μm DWCNT-ADC formed a separate branch within the ADC cluster, indicating modest molecular divergence, possibly influenced by differences in the CNT fiber length or structural characteristics of the agglomerates formed by the DWCNTs. The results of the comparative pathway analysis between ADCs and MM, along with their key characteristics, are described in the following section.

### 3.3. Comparative Pathway Analysis of Adenocarcinomas (ADCs) and Malignant Mesothelioma (MM)

#### 3.3.1. Commonly Dysregulated Canonical Pathways in All MWCNT-Induced ADCs and MM

Canonical pathway analysis revealed a set of six canonical pathways consistently downregulated in all four cancer types: MWCNT-MM and ADCs induced by 1.5 μm DWCNT, 7 μm DWCNT, and MWCNT-7 (Table 1). These pathways represent common deregulation in both histological cancer subtypes, MM and ADC, induced by different types of MWCNTs.

#### 3.3.2. Uniquely Dysregulated Canonical Pathways in MWCNT-7-Induced Malignant Mesothelioma (MM)

As shown in the hierarchical clustering analysis (Figure 2), MWCNT-7-MM exhibited a distinct molecular profile characterized by 26 uniquely upregulated and 11 uniquely downregulated canonical pathways that were not observed in any of the three ADCs (Table S3). The upregulated pathways were predominantly associated with extracellular matrix (ECM) remodeling, fibrosis, and structural reorganization, including extracellular matrix organization, collagen biosynthesis and modifying enzymes, collagen degradation, collagen chain trimerization, actin cytoskeleton signaling, and pulmonary fibrosis idiopathic signaling. These changes indicate a strong fibrotic and ECM-driven tumor phenotype.

In contrast, the downregulated pathways included the Th1 pathway, neutrophil degranulation, neutrophil extracellular trap signaling, and surfactant metabolism, suggesting the suppression of innate immune function and lung-specific physiological responses. Collectively, these uniquely deregulated pathways highlight the fibrotic, immunosuppressive nature of MM and support the notion that MWCNT-induced mesothelioma arises through distinct biological mechanisms compared to MWCNT-induced adenocarcinomas.

#### 3.3.3. Uniquely Dysregulated Canonical Pathways in Adenocarcinomas (ADCs)

Canonical pathway analysis revealed 1 uniquely upregulated and 13 uniquely downregulated canonical pathways across all three MWCNT-induced ADCs, which were not observed in the MWCNT-7-MM (Table 2). The deregulation of these pathways in ADCs but not in MM highlights the distinct molecular profiles and provides insight into the shared biological characteristics of CNT-induced lung ADCs.

#### 3.3.4. Comparison of Canonical Pathways Among MWCNT-Induced Adenocarcinomas (ADCs)

Pathway analyses also revealed distinct molecular differences among CNT-induced ADCs (Table S4). Notably, 7 μm DWCNT-ADC and MWCNT-7 ADC shared a high number of commonly deregulated pathways, both upregulated and downregulated, which were absent in 1.5 μm DWCNT-ADC. For example, pathways such as PTEN Signaling, PPAR Signaling, Pulmonary Healing Signaling Pathway, and several immune- and signaling-related pathways (e.g., Oxytocin Signaling, NOTCH2, VEGF Signaling, and Pulmonary Fibrosis Idiopathic Signaling) were commonly upregulated or downregulated in MWCNT-7-ADC and 7 μm DWCNT-ADC but not altered in 1.5 μm DWCNT-ADC. This trend was also reflected in hierarchical clustering analysis (Figure 2), where 7 μm DWCNT-ADC clustered closely with MWCNT-7-ADC, as described above.

In contrast, 1.5 μm DWCNT-ADC showed a unique expression profile, with several pathways specifically upregulated (TGF-β Signaling, Apelin Adipocyte Signaling, Glycolysis I) or downregulated (PI3K Cascade, Semaphorin Interactions) only in 1.5 μm DWCNT-ADC. These differences suggest that 1.5 μm DWCNT-ADC diverges molecularly from the other two ADCs. Thus, while all three ADC tumors exhibited some commonly upregulated or downregulated pathways, 7 μm DWCNT-ADC showed greater molecular similarity to MWCNT-7-ADC than to 1.5 μm DWCNT-ADC. Since the only difference between 1.5 µm DWCNT and 7 µm DWCNT is the length of the fiber, this suggests that the differences between 1.5 μm DWCNT-ADC and 7 μm DWCNT-ADC were likely due to the agglomerates formed by these DWCNTs.

#### 3.3.5. Identification of Specific Biomarker Candidates for Adenocarcinomas (ADCs) and Malignant Mesothelioma (MM)

Given that upregulated genes are more readily detectable, more practical for clinical testing, and generally have greater potential as biomarkers than downregulated genes, only upregulated DEGs were considered as biomarker candidates in the present study. ADC-specific biomarker candidates were identified from the 81 genes consistently upregulated across all the ADCs induced by the three different types of MWCNT but not differentially expressed in MWCNT-7-MM. Conversely, MM-specific biomarker candidates were selected from 432 genes upregulated exclusively in the MM tumor and not differentially expressed in any of the ADC tumors.

A total of 11 genes were identified as ADC-specific biomarker candidates, most of which are localized to the extracellular space or plasma membrane, supporting their potential utility in non-invasive diagnostics using serum or bronchoalveolar lavage fluid (Table 3). Among these, complement factor I (CFI) and secreted phosphoprotein 1 (SPP1) exhibited notably high expression levels across all ADC tumors, underscoring their diagnostic relevance. CFI showed fold-changes of 162, 202, and 36 in 1.5 μm DWCNT, 7 μm DWCNT, and MWCNT-7-ADCs, respectively. SPP1 exhibited expression values of 147 and 138 and a 9-fold increase in the corresponding tumors.

Among the DEGs specifically upregulated in MWCNT-7-MM, only fibronectin 1 (FN1) was identified as a potential diagnostic biomarker, with a 10-fold increase compared to controls (Table 3).

## 4. Discussion

Despite growing experimental evidence and concern regarding the carcinogenic potential of MWCNTs, the molecular mechanisms underlying MWCNT-induced lung tumorigenesis remain poorly understood. This study is the first to present a comparative microarray-based gene expression analysis of adenocarcinomas (ADCs) induced by three structurally distinct CNTs, 1.5 μm DWCNT, 7 μm DWCNT, and MWCNT-7, and of a malignant mesothelioma (MM) induced by MWCNT-7, as no prior studies have reported gene expression profiling of CNT-induced tumors. By identifying both distinct and shared DEGs and deregulated canonical pathways across tumor types, we demonstrate that the gene expression profile of MWCNT-7-induced MM is clearly distinct from that of ADCs. In addition, while all three ADC tumors exhibited overlapping deregulated pathways and clustered together, 7 μm DWCNT-ADC displayed greater molecular similarity to MWCNT-7-ADC than to 1.5 μm DWCNT-ADC.

In MWCNT-7-MM, 26 canonical pathways were uniquely upregulated, and 11 were downregulated—none of which were observed in the ADC tumors. The upregulated pathways were predominantly associated with ECM remodeling, fibrotic activation, and cytoskeletal reorganization—hallmarks of MM pathology. Notably, the activation of idiopathic pulmonary fibrosis signaling, actin cytoskeleton signaling, ECM organization, collagen biosynthesis and degradation, collagen trimerization, and calcium signaling suggested a strong fibrogenic response and a remodeled tumor microenvironment [26,27]. These features highlight the critical role of ECM stiffness and remodeling in tumor progression [28,29,30,31]. In addition, MM showed the selective downregulation of immune- and lung-specific pathways, such as Th1 signaling, neutrophil degranulation, neutrophil extracellular trap (NET) formation, and surfactant metabolism, indicating impaired innate immunity and disrupted pulmonary homeostasis [32,33,34]. Collectively, these uniquely deregulated pathways in MWCNT-7-MM support a fibrotic, immunosuppressive, and ECM-driven tumor phenotype, distinct from the molecular characteristics of CNT-induced ADCs.

Thirteen canonical pathways were commonly downregulated across all MWCNT-induced ADC tumors but not in MM, further highlighting their molecular divergence. Among these were the RHO GTPase Cycle, which regulates cell migration and invasion and is frequently altered in lung ADC [35]. The downregulation of the role of macrophages, fibroblasts, and endothelial cells in rheumatoid arthritis pathway indicates dysregulated stromal–immune interactions. The downregulation of the acetylcholine receptor signaling pathway and serotonin receptor signaling pathway has been linked to tumor proliferation, survival, and immune modulation [36,37], and the downregulation of the glutamatergic receptor signaling pathway is associated with tumor growth and chemoresistance [38,39]. Additionally, GPCR-related pathways—GPCR-mediated nutrient sensing, G beta gamma signaling, and G alpha (q) signaling events—were downregulated, potentially indicating impaired GPCR-mediated regulation of tumor behavior [40,41]. Collectively, these consistently downregulated pathways across the ADCs point to shared molecular features driven by MWCNT exposure and emphasize the unique carcinogenic profile of MWCNT-7-MM—marked by ECM remodeling, fibrosis, and immune suppression.

In addition to pathway-level differences, we identified several candidate biomarkers that may distinguish ADC from MM, including CFI and SPP1. CFI is a serine protease that regulates the complement system by degrading C4b and C3b, thereby limiting complement activation [42]. The overexpression of CFI is associated with tumor progression and poor prognosis in several cancers [43,44,45,46], including non-small cell lung cancer [47], likely via immune evasion [45] and tumor cell proliferation, migration, and invasion [44]. SPP1 (also known as osteopontin) is a multifunctional secreted glycoprotein broadly recognized as a marker of poor prognosis in numerous cancers [48,49], including lung cancer [50,51,52]. It promotes immune evasion, enhances tumor-associated fibrosis, and facilitates metastasis [48,51].

In contrast, FN1 was identified as an MM-specific diagnostic marker. FN1 encodes a key ECM protein involved in cell adhesion and migration. Its overexpression has been linked to poor prognosis in gastric, ovarian, and breast cancers [53,54,55,56,57]. CF1, SSP1, and FN1 encode extracellular proteins that were highly overexpressed in the tumor samples analyzed, suggesting their potential utility as non-invasive diagnostic biomarkers. Further experimental and clinical validation—especially in combination with proteomic profiling—will be essential to assess their diagnostic specificity and sensitivity.

While direct extrapolation to human occupational exposure is challenging due to limited exposure data, it is noteworthy that the National Institute for Occupational Safety and Health (NIOSH) has established a Recommended Exposure Limit (REL) of 1 µg/m^3^ (8 h time-weighted average) for carbon nanotubes and nanofibers [58]. This value was derived from subchronic inhalation studies in rats, based on an NOAEL corresponding to a lung burden of 11.7 µg/lung [59] and an LOAEL corresponding to 16.0 µg/lung [60]. In the present study, the total doses administered per rat were 50.4 μg for 1.5 µm DWCNT, 232 μg for 7 µm DWCNT, and 504 μg for MWCNT-7. These exposure levels are substantially higher than those used to estimate the REL for humans and were selected to ensure tumor development for mechanistic evaluation within the experimental timeframe.

Several limitations of this study should be noted. First, only a single snap-frozen tumor sample per MWCNT-induced cancer type was analyzed for each MWCNT-induced cancer type, raising the possibility that the findings may be influenced by both intra-tumoral and inter-tumoral heterogeneity. Importantly, when analyzing single tumors, background mutations that do not affect carcinogenesis will be detected. The detection of background mutations such as DEGs can also affect pathway analysis. Second, z-scores were used to define differentially expressed genes. In future studies, the functional validation of the effect of these identified up- and downregulated genes needs to be carried out. Third, to distinguish differences driven by tumor origin from those attributable to specific MWCNT types, it is essential to analyze several biologically independent tumors. Follow-up tumor induction is currently underway, and analyses will include comprehensive histopathological and immunohistochemical evaluations of key molecular markers reported in the present study. These efforts aim to more precisely characterize and differentiate all lung tumors from the TIPS carcinogenesis study and to enable a more precise comparison of tumor subtypes and their underlying molecular mechanisms. Fourth, although several candidate biomarkers were identified, their diagnostic and prognostic utility requires confirmation in future studies using biofluids such as serum, bronchoalveolar lavage fluid, and matched lung tissue samples from both animal models and human cohorts. Moreover, the extrapolation of these results to human disease remains uncertain. There are currently no confirmed clinical cases of MWCNT-induced MM or ADC in humans. Thus, while the gene expression profiles presented here provide valuable insight into the carcinogenic mechanisms of MWCNTs in experimental settings, their direct relevance to human risk assessment and diagnosis should be interpreted with caution. In addition, validation in independent tumor samples and human cohorts is essential to confirm biomarker utility.

In conclusion, our findings highlight the significant influence of MWCNT fiber size and structure on the nature of carcinogenic responses. The identification of MM- and ADC-specific molecular signatures offers potential utility in distinguishing between these histological subtypes and contributes to the development of novel diagnostic or predictive biomarkers. While additional validation is warranted, these results advance our understanding of the molecular landscape of MWCNT-induced carcinogenesis and underscore the importance of fiber-dependent differences in tumor biology. In addition, these findings support the conclusion that the lung ADCs that developed in the rats administered DWCNTs reported in our companion long-term carcinogenicity study did not develop spontaneously but were induced by the administered DWCNTs. Our results also support the conclusions that while thin flexible CNTs are not carcinogenic in the pleural cavity, they are carcinogenic in the lung [5,6,11,16,17], highlighting the need for further evaluation of the carcinogenicity of inhaled CNTs.

## Figures and Tables

**Table 1:** Canonical pathways downregulated in all four cancers.

	MWCNT-7-MM	1.5 μm DWCNT-ADC	MWCNT-7-ADC	7 μm DWCNT-ADC
**Pathways Commonly Downregulated in All Cancers**				
Natural Killer Cell Signaling	−2.5	−3.5	−2.8	−3.2
Immunoregulatory Interactions Between a Lymphoid and a Non-Lymphoid Cell	−2.3	−2.3	−2.7	−3.5
Breast Cancer Regulation by Stathmin1	−2.2	−3.3	−3.0	−4.6
BBSome Signaling Pathway	−2.6	−2.9	−2.3	−3.4
FAK Signaling	−2.5	−3.4	−4.0	−4.6
Adrenomedullin signaling pathway	−2.1	−2.1	−3.2	−2.7

The values listed are z scores.

**Table 2:** Canonical pathways uniquely deregulated in all MWCNT-induced ADCs.

	MWCNT-7-MM	1.5 μm DWCNT-ADC	MWCNT-7-ADC	7 μm DWCNT-ADC
**Pathways Uniquely Upregulated in All 3 ADCs**				
Sleep NREM Signaling Pathway	-	2.2	2.6	2.3
**Pathways Uniquely Downregulated in All 3 ADCs**				
RHO GTPase Cycle	-	−2.2	−3.0	−2.9
Role of Macrophages, Fibroblasts, and Endothelial Cells in Rheumatoid Arthritis	-	−2.3	−2.9	−4.0
Acetylcholine Receptor Signaling Pathway	-	−2.7	−2.5	−2.0
Neuropathic Pain Signaling in Dorsal Horn Neurons	-	−2.0	−2.6	−2.1
Serotonin Receptor Signaling	-	−2.0	−3.2	−2.7
Glutaminergic Receptor Signaling Pathway (Enhanced)	-	−2.1	−2.6	−2.7
GPCR-Mediated Nutrient Sensing in Enteroendocrine Cells	-	−2.4	−2.7	−2.3
G Beta Gamma Signaling	-	−2.0	−2.9	−2.3
G Alpha (q) Signaling Events	-	−2.3	−2.9	−2.3
eNOS Signaling	-	−2.1	−3.0	−2.8
Smooth Muscle Contraction	-	−2.4	−2.4	−2.6
Relaxin Signaling	-	−2.0	−3.2	−2.9
Synaptic Long-Term Depression	-	−2.3	−3.0	−3.2

The values listed are z scores. “-” indicates that the pathway was not significantly upregulated or downregulated in that tumor model.

**Table 3:** Tumor-type-specific biomarker candidates for ADC and MM.

Symbol	Entrez Gene Name	Location	Family	Fold Changes (vs. Controls) *			
				1.5 μm DWCNT-ADC	7 μm DWCNT-ADC	MWCNT-7- ADC	MWCNT-7-MM
**ADC-specific biomarker candidates**							
CFI	Complement factor I	Extracellular space	Peptidase	162	202	36	-
SPP1	Secreted phosphoprotein 1	Extracellular space	Cytokine	147	138	9	-
BPIFB2	BPI fold-containing family B member 2	Extracellular space	Other	91	137	5	-
SERPINF1	Serpin family F member 1	Extracellular space	Other	80	8	4	-
DRP2	Dystrophin-related protein 2	Plasma membrane	Other	28	15	23	-
SERPINE2	Serpin family E member 2	Extracellular space	Other	19	28	17	-
CA3	Carbonic anhydrase 3	Cytoplasm	Enzyme	17	6	5	-
LAMB3	Laminin subunit beta 3	Extracellular space	Transporter	14	15	9	-
PDE4C	Phosphodiesterase 4C	Cytoplasm	Enzyme	11	13	12	-
BPIFB1	BPI fold-containing family B member 1	Extracellular space	Other	7	10	4	-
SLC3A1	Solute carrier family 3 member 1	Plasma membrane	Transporter	5	8	12	-
**MM-specific biomarker candidate**							
FN1	Fibronectin 1	Extracellular space	Other	-	-	-	10

* Values represent fold changes relative to controls. “-” indicates no significant change (fold change < 2 and/or |z-score| < 2).

## Data Availability

The raw data supporting the conclusions of this article will be made available by the authors upon request.

## Supplementary Materials

The following supporting information can be downloaded at https://www.mdpi.com/article/10.3390/nano15231806/s1. Figure S1: Hierarchical clustering of canonical pathways enriched in MWCNT-induced ADCs and MM, with pathway names annotated; Table S1: DEGs common to all three ADCs: 1.5 μm DWCNT-ADC, 7 μm DWCNT-ADC, and MWCNT-7-ADC; Table S2: DEGs uniquely identified in MWCNT-7-MM and not detected in any ADCs; Table S3: Canonical pathways uniquely dysregulated in MWCNT-Induced MM; Table S4: Comparison of canonical pathways enriched across MWCNT-induced ADCs.

## Abbreviations

ADC	Adenocarcinoma
CFI	Complement factor I
CNTs	Carbon nanotubes
DEGs	Differentially expressed genes
ECM	Extracellular matrix
FN1	Fibronectin 1
GPCR	G protein-coupled receptor
IKB	Ingenuity Knowledge Base
MM	Malignant mesothelioma
MWCNT	Multi-walled CNT
MWCNT-7	Multi-walled carbon nanotube-7
NET	Neutrophil extracellular trap
SPP1	Secreted phosphoprotein 1
SWCNT	Single-walled carbon nanotube
TIPS	Intra-tracheal intrapulmonary spraying
