# Supplementary material for: Comparative Gene Expression Analysis of Malignant Mesothelioma and Lung Adenocarcinomas Induced by Multi-Walled Carbon Nanotube-7 and Double-Walled Carbon Nanotubes in Rats: Distinct Molecular Signatures and Canonical Pathways
Source: Nanomaterials (Basel). 2025 Nov 29;15(23):1806. doi: 10.3390/nano15231806 (PMC12693446; doi:10.3390/nano15231806)
Supplement: Supplementary file 1 [file nanomaterials-15-01806-s001.zip › nanomaterials-3963728-supplementary.pdf]

Supplementary Materials

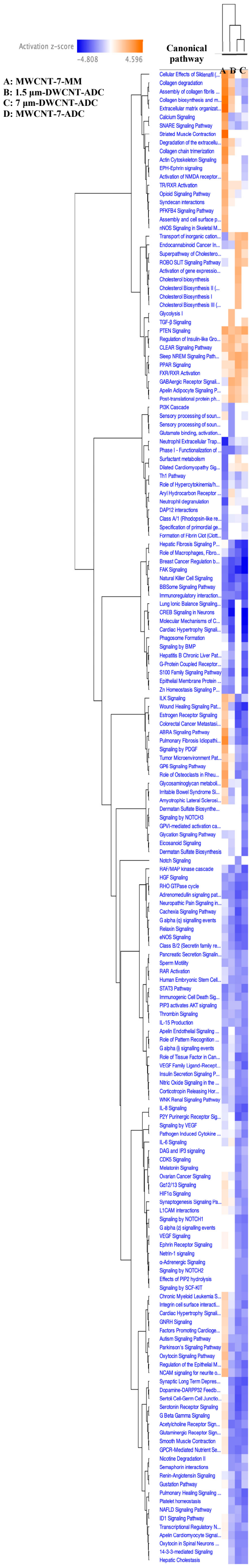

Figure S1. Hierarchical clustering of canonical pathways enriched in MWCNT-Induced ADCs and MM, with pathway names annotated.

Supplementary Table S1

Table S1. DEGs Common to All Three ADCs: 1.5 μm-DWCNT-ADC, 7 μm-DWCNT-ADC, and MWCNT-7-ADC.

		Fold changes (vs controls)			
**Symbol**	**Entrez Gene Name**	**1.5 μm-DWCNT-ADC**	**7 μm-DWCNT-ADC**	**MWCNT-7-ADC**	**Location**
MUC5B	mucin 5B, oligomeric mucus/gel-forming	1363	341	17	Extracellular Space
Retnla	resistin like alpha	703	1822	3859	Extracellular Space
SLC26A4	solute carrier family 26 member 4	634	127	99	Plasma Membrane
CYSLTR1	cysteinyl leukotriene receptor 1	240	5	7	Plasma Membrane
EGLN3	egl-9 family hypoxia inducible factor 3	212	37	6	Cytoplasm
TAC4	tachykinin precursor 4	192	55	100	Extracellular Space
CFI	complement factor I	162	202	36	Extracellular Space
SPP1	secreted phosphoprotein 1	147	138	9	Extracellular Space
ELF5	E74 like ETS transcription factor 5	110	15	17	Nucleus
BPIFB2	BPI fold containing family B member 2	91	137	5	Extracellular Space
SERPINF1	serpin family F member 1	80	8	4	Extracellular Space
SERPINE1	serpin family E member 1	70	15	7	Extracellular Space
SLC1A2	solute carrier family 1 member 2	68	16	4	Plasma Membrane
Kng1/Kng2	kininogen 2	66	79	39	Extracellular Space
EHF	ETS homologous factor	65	14	13	Nucleus
PF4	platelet factor 4	61	8	6	Extracellular Space
LBP	lipopolysaccharide binding protein	54	17	28	Plasma Membrane
ADIPOQ	adiponectin, C1Q and collagen domain containing	49	23	10	Extracellular Space
BHLHE41	basic helix-loop-helix family member e41	49	8	10	Nucleus
Nipsnap3b	nipsnap homolog 3B	44	3	2	Cytoplasm
BMP5	bone morphogenetic protein 5	43	20	17	Extracellular Space
PTGES	prostaglandin E synthase	42	14	4	Cytoplasm
CPNE5	copine 5	41	29	21	Plasma Membrane
MC5R	melanocortin 5 receptor	38	24	10	Plasma Membrane
LRRN4	leucine rich repeat neuronal 4	37	-6	-5	Plasma Membrane
MUC1	mucin 1, cell surface associated	35	19	27	Plasma Membrane
Dlg5l12 (includes others)	discs large MAGUK scaffold protein 5 like 12	34	6	2	Other
MIF	macrophage migration inhibitory factor	31	7	3	Extracellular Space
ASS1	argininosuccinate synthase 1	30	13	5	Cytoplasm
DRP2	dystrophin related protein 2	28	15	23	Plasma Membrane
MMP12	matrix metallopeptidase 12	28	30	13	Extracellular Space
TSPAN1	tetraspanin 1	24	12	8	Cytoplasm
Fcrl2	Fc receptor-like 2	23	3	5	Plasma Membrane
CILP	cartilage intermediate layer protein	21	8	5	Extracellular Space
VSIG2	V-set and immunoglobulin domain containing 2	20	3	3	Plasma Membrane
SERPINE2	serpin family E member 2	19	28	17	Extracellular Space
CA3	carbonic anhydrase 3	17	6	5	Cytoplasm
SLC7A7	solute carrier family 7 member 7	16	9	10	Plasma Membrane
S100A9	S100 calcium binding protein A9	14	-8	-13	Cytoplasm
BMP3	bone morphogenetic protein 3	14	23	34	Extracellular Space
Mt1	metallothionein 1	14	71	23	Cytoplasm
LAMB3	laminin subunit beta 3	14	15	9	Extracellular Space
CLDN3	claudin 3	14	21	25	Plasma Membrane
INHBB	inhibin subunit beta B	12	7	8	Extracellular Space
CYGB	cytoglobin	12	3	3	Cytoplasm
PARM1	prostate androgen-regulated mucin-like protein 1	12	11	18	Extracellular Space
CD86	CD86 molecule	12	9	6	Plasma Membrane
PDE4C	phosphodiesterase 4C	11	13	12	Cytoplasm
MANSC1	MANSC domain containing 1	11	9	7	Other
LAD1	ladinin 1	10	14	10	Extracellular Space
HMOX1	heme oxygenase 1	10	12	9	Cytoplasm
TNFRSF21	TNF receptor superfamily member 21	9	9	9	Plasma Membrane
MC2R	melanocortin 2 receptor	8	25	14	Plasma Membrane
CDH3	cadherin 3	8	6	8	Plasma Membrane
HLA-DMA	major histocompatibility complex, class II, DM alpha	8	13	13	Plasma Membrane
MAPK13	mitogen-activated protein kinase 13	8	11	12	Cytoplasm

CLDN7	claudin 7	8	6	5	Plasma Membrane
ACP5	acid phosphatase 5, tartrate resistant	8	9	9	Cytoplasm
FOXQ1	forkhead box Q1	7	12	8	Nucleus
PIRT	phosphoinositide interacting regulator of transient receptor potential channels	7	30	43	Plasma Membrane
Slc23a4	solute carrier family 23 member 4	7	11	6	Other
BPIFB1	BPI fold containing family B member 1	7	10	4	Extracellular Space
ESR1	estrogen receptor 1	7	8	7	Nucleus
ST14	ST14 transmembrane serine protease matriptase	6	6	5	Plasma Membrane
TMEM54	transmembrane protein 54	6	48	20	Other
Gm11545	predicted gene 11545	6	4	5	Other
CX3CL1	C-X3-C motif chemokine ligand 1	6	9	10	Extracellular Space
CTHRC1	collagen triple helix repeat containing 1	6	9	7	Extracellular Space
KCTD16	potassium channel tetramerization domain containing 16	6	6	3	Plasma Membrane
AC107446.1	Sproutin	6	5	4	Other
PEX11A	peroxisomal biogenesis factor 11 alpha	6	8	4	Cytoplasm
SLC3A1	solute carrier family 3 member 1	5	8	12	Plasma Membrane
LOC100362296/S100a11l2	S100 calcium binding protein A11 like 2	5	4	5	Other
ELF3	E74 like ETS transcription factor 3	4	5	5	Nucleus
DEGS2	delta 4-desaturase, sphingolipid 2	4	9	4	Cytoplasm
ENTPD3	ectonucleoside triphosphate diphosphohydrolase 3	4	3	2	Plasma Membrane
OLFML2B	olfactomedin like 2B	4	3	2	Extracellular Space
SLAMF9	SLAM family member 9	3	5	3	Extracellular Space
AREG	amphiregulin	3	7	6	Extracellular Space
GABRR2	gamma-aminobutyric acid type A receptor subunit rho2	3	3	4	Plasma Membrane
OTX1	orthodenticle homeobox 1	3	7	4	Nucleus
SLC16A14	solute carrier family 16 member 14	3	3	4	Other
SLAMF8	SLAM family member 8	3	4	4	Extracellular Space
DNAI7	dynein axonemal intermediate chain 7	-2	-2	-2	Cytoplasm
PGLYRP1	peptidoglycan recognition protein 1	-2	-2	-2	Plasma Membrane
SNTN	sentan, cilia apical structure protein	-2	-3	-2	Extracellular Space
OAS2	2'-5'-oligoadenylate synthetase 2	-2	-3	-3	Cytoplasm
RUSC2	RUN and SH3 domain containing 2	-2	-3	-3	Cytoplasm
FAM47E	family with sequence similarity 47 member E	-2	-2	-3	Cytoplasm
NOVA1	NOVA alternative splicing regulator 1	-2	-3	-3	Nucleus
AMOT	angiomotin	-2	-3	-3	Plasma Membrane
CFAP52	cilia and flagella associated protein 52	-2	-3	-2	Cytoplasm
ABCA8	ATP binding cassette subfamily A member 8	-2	-2	-2	Plasma Membrane
FILIP1L	filamin A interacting protein 1 like	-2	-2	-2	Nucleus
Tarm1	T cell-interacting, activating receptor on myeloid cells 1	-2	-2	-3	Plasma Membrane
PON1	paraoxonase 1	-2	-2	-2	Extracellular Space
SYDE1	synapse defective Rho GTPase homolog 1	-2	-2	-2	Cytoplasm
MFSD4A	major facilitator superfamily domain containing 4A	-2	-3	-2	Other
Tcp11x2	t-complex 11 family, X-linked 2	-2	-3	-3	Other
DMC1	DNA meiotic recombinase 1	-2	-2	-2	Nucleus
SYTL3	synaptotagmin like 3	-2	-2	-2	Cytoplasm
ZBTB46	zinc finger and BTB domain containing 46	-2	-2	-2	Nucleus
CORO2B	coronin 2B	-2	-3	-3	Plasma Membrane
CYTL1	cytokine like 1	-2	-3	-3	Extracellular Space
TRARG1	trafficking regulator of GLUT4 (SLC2A4) 1	-2	-2	-2	Plasma Membrane
HPGDS	hematopoietic prostaglandin D synthase	-2	-3	-3	Cytoplasm
C2orf88	chromosome 2 open reading frame 88	-2	-3	-3	Other
Grap	GRB2-related adaptor protein	-2	-3	-3	Other
GPR50	G protein-coupled receptor 50	-2	-2	-2	Plasma Membrane
ZPBP2	zona pellucida binding protein 2	-2	-3	-2	Extracellular Space
NR4A3	nuclear receptor subfamily 4 group A member 3	-2	-2	-2	Nucleus
SRGAP3	SLIT-ROBO Rho GTPase activating protein 3	-2	-4	-3	Cytoplasm
CLEC1B	C-type lectin domain family 1 member B	-2	-3	-2	Plasma Membrane
ERBB4	erb-b2 receptor tyrosine kinase 4	-2	-3	-3	Plasma Membrane
CD247	CD247 molecule	-2	-3	-2	Plasma Membrane
TMEM117	transmembrane protein 117	-2	-2	-2	Cytoplasm
CCN5	cellular communication network factor 5	-2	-2	-2	Extracellular Space
PLPP4	phospholipid phosphatase 4	-3	-2	-2	Plasma Membrane
CYP26B1	cytochrome P450 family 26 subfamily B member 1	-3	-3	-3	Cytoplasm
ADRA1A	adrenoceptor alpha 1A	-3	-3	-3	Plasma Membrane
C1QTNF7	C1q and TNF related 7	-3	-3	-2	Extracellular Space
Ly49i2 (includes others)	Ly49 inhibitory receptor 2	-3	-2	-2	Other
HOXA6	homeobox A6	-3	-4	-3	Nucleus
OPCML	opioid binding protein/cell adhesion molecule like	-3	-2	-2	Plasma Membrane
CHRNE	cholinergic receptor nicotinic epsilon subunit	-3	-3	-3	Plasma Membrane
DNAH5	dynein axonemal heavy chain 5	-3	-2	-2	Cytoplasm
SCEL	sciellin	-3	-3	-3	Cytoplasm
TBC1D10C	TBC1 domain family member 10C	-3	-2	-2	Nucleus
LAT2	linker for activation of T cells family member 2	-3	-3	-3	Plasma Membrane

Ace	angiotensin I converting enzyme	-3	-13	-10	Plasma Membrane
HDX	highly divergent homeobox	-3	-2	-3	Nucleus
LRRC23	leucine rich repeat containing 23	-3	-2	-3	Plasma Membrane
OTUD7A	OTU deubiquitinase 7A	-3	-3	-3	Cytoplasm
UBXN10	UBX domain protein 10	-3	-3	-2	Extracellular Space
ADAMTSL2	ADAMTS like 2	-3	-3	-3	Extracellular Space
GIMAP5	GTPase, IMAP family member 5	-3	-4	-3	Cytoplasm
ADAMTS8	ADAM metallopeptidase with thrombospondin type 1 motif 8	-3	-3	-2	Extracellular Space
ARID3B	AT-rich interaction domain 3B	-3	-3	-2	Nucleus
KLF12	KLF transcription factor 12	-3	-3	-2	Nucleus
SLC26A3	solute carrier family 26 member 3	-3	-3	-3	Plasma Membrane
LRRN3	leucine rich repeat neuronal 3	-3	-2	-2	Extracellular Space
Apol11a	apolipoprotein L11a	-3	-3	-3	Other
ZNF697	zinc finger protein 697	-3	-3	-4	Other
SGIP1	SH3GL interacting endocytic adaptor 1	-3	-3	-3	Cytoplasm
DNAH7	dynein axonemal heavy chain 7	-3	-3	-4	Extracellular Space
PPP1R9A	protein phosphatase 1 regulatory subunit 9A	-3	-3	-3	Plasma Membrane
ZNF683	zinc finger protein 683	-3	-3	-4	Other
PDE8B	phosphodiesterase 8B	-3	-3	-3	Cytoplasm
EFHC2	EF-hand domain containing 2	-3	-3	-3	Cytoplasm
DNAH3	dynein axonemal heavy chain 3	-3	-3	-3	Extracellular Space
IQUB	IQ motif and ubiquitin domain containing	-3	-3	-3	Cytoplasm
TSGA10	testis specific 10	-3	-3	-3	Cytoplasm
CHIC1	cysteine rich hydrophobic domain 1	-3	-3	-4	Plasma Membrane
AGBL3	AGBL carboxypeptidase 3	-3	-3	-3	Cytoplasm
ANKRD33B	ankyrin repeat domain 33B	-3	-3	-3	Other
PRKCQ	protein kinase C theta	-3	-3	-2	Cytoplasm
DMD	dystrophin	-3	-2	-3	Plasma Membrane
RIPPLY3	ripply transcriptional repressor 3	-3	-3	-3	Nucleus
PACRG	parkin coregulated	-3	-2	-2	Nucleus
GPR52	G protein-coupled receptor 52	-3	-4	-4	Plasma Membrane
FBXL13	F-box and leucine rich repeat protein 13	-3	-3	-3	Cytoplasm
Gimap7	GTPase, IMAP family member 7	-3	-4	-3	Cytoplasm
SAXO2	stabilizer of axonemal microtubules 2	-3	-3	-3	Nucleus
VWA3A	von Willebrand factor A domain containing 3A	-3	-3	-4	Other
GPR171	G protein-coupled receptor 171	-4	-3	-3	Plasma Membrane
Aldh1a7	aldehyde dehydrogenase family 1, subfamily A7	-4	-2	-3	Cytoplasm
KCNB1	potassium voltage-gated channel subfamily B member 1	-4	-3	-4	Plasma Membrane
CLSTN2	calsyntenin 2	-4	-3	-3	Plasma Membrane
CCDC39	coiled-coil domain 39 molecular ruler complex subunit	-4	-3	-3	Cytoplasm
STX11	syntaxin 11	-4	-3	-4	Plasma Membrane
ZBBX	zinc finger B-box domain containing	-4	-4	-3	Other
RSAD2	radical S-adenosyl methionine domain containing 2	-4	-3	-3	Cytoplasm
SAMD3	sterile alpha motif domain containing 3	-4	-5	-4	Other
Zfp605l1	zinc finger protein 605 like 1	-4	-4	-3	Other
Tex16	testis expressed gene 16	-4	-3	-4	Other
MARK1	microtubule affinity regulating kinase 1	-4	-5	-4	Cytoplasm
BACH2	BTB domain and CNC homolog 2	-4	-3	-3	Nucleus
LPAR3	lysophosphatidic acid receptor 3	-4	-3	-2	Plasma Membrane
CNN1	calponin 1	-4	-4	-3	Cytoplasm
NELL1	neural EGFL like 1	-5	-6	-6	Extracellular Space
CFAP65	cilia and flagella associated protein 65	-5	-4	-4	Cytoplasm
CDHR1	cadherin related family member 1	-5	-5	-4	Plasma Membrane
CCM2L	CCM2 like scaffold protein	-5	-6	-5	Other
METTL24	methyltransferase like 24	-5	-5	-4	Other
MCTP1	multiple C2 and transmembrane domain containing 1	-5	-5	-4	Cytoplasm
RGS7	regulator of G protein signaling 7	-5	-7	-6	Cytoplasm
PTPN14	protein tyrosine phosphatase non-receptor type 14	-5	-6	-6	Cytoplasm
CFAP54	cilia and flagella associated protein 54	-5	-4	-5	Cytoplasm
TNIK	TRAF2 and NCK interacting kinase	-5	-5	-6	Plasma Membrane
NETO2	neuropilin and tolloid like 2	-5	-5	-5	Plasma Membrane
GIMAP6	GTPase, IMAP family member 6	-5	-6	-5	Cytoplasm
ADHFE1	alcohol dehydrogenase iron containing 1	-5	-4	-4	Cytoplasm
DNAH9	dynein axonemal heavy chain 9	-5	-5	-5	Cytoplasm
SLC28A2	solute carrier family 28 member 2	-5	-8	-12	Plasma Membrane
Col4a4	collagen type IV alpha 4 chain	-5	-6	-6	Extracellular Space
SOX17	SRY-box transcription factor 17	-5	-8	-7	Nucleus
ACKR3	atypical chemokine receptor 3	-5	-12	-11	Plasma Membrane
FAT4	FAT atypical cadherin 4	-5	-16	-9	Cytoplasm
CACNB2	calcium voltage-gated channel auxiliary subunit beta 2	-5	-6	-5	Plasma Membrane
ZNF462	zinc finger protein 462	-5	-4	-6	Nucleus
TMCC2	transmembrane and coiled-coil domain family 2	-5	-4	-5	Extracellular Space
Gzmc	granzyme C	-6	-8	-14	Other
CFAP47	cilia and flagella associated protein 47	-6	-5	-6	Cytoplasm
SPEF2	sperm flagellar 2	-6	-6	-6	Cytoplasm

KANK3	KN motif and ankyrin repeat domains 3	-6	-7	-8	Cytoplasm
KLRG1	killer cell lectin like receptor G1	-6	-5	-5	Plasma Membrane
DNAI3	dynein axonemal intermediate chain 3	-6	-6	-5	Cytoplasm
WFDC1	WAP four-disulfide core domain 1	-6	-6	-8	Extracellular Space
PLCE1	phospholipase C epsilon 1	-6	-4	-4	Cytoplasm
ITGAD	integrin subunit alpha D	-6	-5	-4	Plasma Membrane
IL1A	interleukin 1 alpha	-6	-4	-4	Extracellular Space
CHODL	chondrolectin	-6	-6	-4	Plasma Membrane
RGS6	regulator of G protein signaling 6	-6	-5	-7	Cytoplasm
FASLG	Fas ligand	-6	-4	-5	Extracellular Space
STARD9	StAR related lipid transfer domain containing 9	-6	-7	-6	Cytoplasm
BCL6B	BCL6B transcription repressor	-6	-13	-13	Nucleus
EGFL7	EGF like domain multiple 7	-6	-9	-10	Extracellular Space
Gbp6-ps3	guanylate binding protein 6, pseudogene 3	-6	-6	-6	Other
Klre1	killer cell lectin-like receptor family E member 1	-6	-6	-6	Plasma Membrane
Meg3	maternally expressed 3	-6	-9	-9	Nucleus
FZD4	frizzled class receptor 4	-6	-6	-5	Plasma Membrane
SDR9C7	short chain dehydrogenase/reductase family 9C member 7	-6	-7	-5	Other
PLEKHH2	pleckstrin homology, MyTH4 and FERM domain containing H2	-7	-7	-7	Cytoplasm
EPB41L4A	erythrocyte membrane protein band 4.1 like 4A	-7	-8	-8	Extracellular Space
LIMS2	LIM zinc finger domain containing 2	-7	-7	-7	Cytoplasm
SHANK3	SH3 and multiple ankyrin repeat domains 3	-7	-7	-7	Plasma Membrane
GATM	glycine amidinotransferase	-7	-13	-7	Cytoplasm
GATA2	GATA binding protein 2	-7	-8	-7	Nucleus
MAPT	microtubule associated protein tau	-7	-6	-5	Plasma Membrane
Abcb1a	ATP-binding cassette, sub-family B member 1A	-7	-8	-6	Plasma Membrane
SCIN	scinderin	-7	-8	-5	Cytoplasm
LBH	LBH regulator of WNT signaling pathway	-7	-7	-5	Nucleus
MAMDC2	MAM domain containing 2	-7	-7	-4	Extracellular Space
LHFPL3	LHFPL tetraspan subfamily member 3	-7	-8	-7	Other
TRHDE	thyrotropin releasing hormone degrading enzyme	-7	-7	-8	Plasma Membrane
SLC16A12	solute carrier family 16 member 12	-7	-5	-6	Plasma Membrane
PDE3A	phosphodiesterase 3A	-7	-9	-9	Cytoplasm
NCR1	natural cytotoxicity triggering receptor 1	-8	-7	-8	Plasma Membrane
GUCY1B1	guanylate cyclase 1 soluble subunit beta 1	-8	-8	-6	Cytoplasm
HYDIN	HYDIN axonemal central pair apparatus protein	-8	-6	-6	Cytoplasm
NBEA	neurobeachin	-8	-9	-8	Cytoplasm
NIPAL1	NIPA like domain containing 1	-8	-8	-6	Cytoplasm
OMD	osteomodulin	-8	-5	-5	Extracellular Space
FAM174B	family with sequence similarity 174 member B	-8	-6	-6	Other
GUCY1A2	guanylate cyclase 1 soluble subunit alpha 2	-8	-7	-6	Cytoplasm
GRIA3	glutamate ionotropic receptor AMPA type subunit 3	-8	-7	-5	Plasma Membrane
ARL4D	ADP ribosylation factor like GTPase 4D	-8	-8	-5	Nucleus
NEXN	nexilin F-actin binding protein	-8	-8	-6	Plasma Membrane
TXK	TXK tyrosine kinase	-8	-11	-8	Cytoplasm
ADRB2	adrenoceptor beta 2	-8	-8	-8	Plasma Membrane
MYLK	myosin light chain kinase	-8	-8	-5	Cytoplasm
HHIP	hedgehog interacting protein	-8	-6	-10	Plasma Membrane
DLC1	DLC1 Rho GTPase activating protein	-8	-10	-6	Cytoplasm
CLNK	cytokine dependent hematopoietic cell linker	-8	-10	-7	Cytoplasm
EOMES	eomesodermin	-8	-11	-7	Nucleus
SH2D1A	SH2 domain containing 1A	-8	-6	-5	Cytoplasm
KLKB1	kallikrein B1	-9	-4	-5	Extracellular Space
SPON1	spondin 1	-9	-10	-6	Extracellular Space
ADAMTS9	ADAM metallopeptidase with thrombospondin type 1 motif 9	-9	-9	-7	Extracellular Space
ZNF365	zinc finger protein 365	-9	-5	-5	Cytoplasm
JAM2	junctional adhesion molecule 2	-9	-24	-20	Plasma Membrane
Abca8a	ATP-binding cassette, sub-family A member 8a	-9	-7	-6	Plasma Membrane
ZEB1	zinc finger E-box binding homeobox 1	-9	-10	-8	Nucleus
KLF2	KLF transcription factor 2	-9	-8	-9	Nucleus
SLC6A4	solute carrier family 6 member 4	-9	-6	-5	Plasma Membrane
ERG	ETS transcription factor ERG	-9	-18	-11	Nucleus
SCN3B	sodium voltage-gated channel beta subunit 3	-9	-6	-7	Plasma Membrane
SEMA6A	semaphorin 6A	-9	-19	-14	Plasma Membrane
Pcdhgb4	protocadherin gamma subfamily B, 4	-10	-9	-4	Other
PRKCH	protein kinase C eta	-10	-11	-9	Cytoplasm
RGS12	regulator of G protein signaling 12	-10	-10	-8	Nucleus
IL18RAP	interleukin 18 receptor accessory protein	-10	-10	-8	Plasma Membrane
CD93	CD93 molecule	-10	-21	-19	Plasma Membrane
ENG	endoglin	-10	-19	-13	Plasma Membrane
VWF	von Willebrand factor	-10	-17	-13	Extracellular Space
QKI	QKI, KH domain containing RNA binding	-10	-6	-6	Nucleus
CDC14A	cell division cycle 14A	-10	-10	-9	Nucleus
MYZAP	myocardial zonula adherens protein	-10	-13	-9	Plasma Membrane

MUC16	mucin 16, cell surface associated	-10	-14	-15	Plasma Membrane
TSPAN7	tetraspanin 7	-10	-15	-10	Plasma Membrane
CDKL5	cyclin dependent kinase like 5	-10	-7	-10	Nucleus
RAB6B	RAB6B, member RAS oncogene family	-11	-9	-13	Cytoplasm
SLC43A3	solute carrier family 43 member 3	-11	-7	-6	Extracellular Space
Pcdhgb5	protocadherin gamma subfamily B, 5	-11	-6	-7	Other
GSN	gelsolin	-11	-7	-7	Extracellular Space
CRIM1	cysteine rich transmembrane BMP regulator 1	-11	-17	-11	Extracellular Space
RAVER2	ribonucleoprotein, PTB binding 2	-11	-11	-9	Nucleus
BMPR2	bone morphogenetic protein receptor type 2	-11	-6	-7	Plasma Membrane
DNAH12	dynein axonemal heavy chain 12	-11	-8	-10	Cytoplasm
AMIGO2	adhesion molecule with Ig like domain 2	-11	-14	-14	Plasma Membrane
ANXA3	annexin A3	-11	-17	-20	Cytoplasm
KDR	kinase insert domain receptor	-11	-16	-6	Plasma Membrane
GRK5	G protein-coupled receptor kinase 5	-11	-13	-11	Plasma Membrane
NOX4	NADPH oxidase 4	-12	-7	-9	Cytoplasm
SEMA6D	semaphorin 6D	-12	-9	-9	Plasma Membrane
Gbp9 (includes others)	guanylate-binding protein 9	-12	-13	-9	Cytoplasm
PDE5A	phosphodiesterase 5A	-12	-15	-12	Cytoplasm
ARHGAP29	Rho GTPase activating protein 29	-12	-10	-7	Cytoplasm
CFAP44	cilia and flagella associated protein 44	-12	-7	-6	Extracellular Space
Gm12253	predicted gene 12253	-12	-19	-18	Plasma Membrane
RGCC	regulator of cell cycle	-12	-11	-6	Other
Rgccl1	regulator of cell cycle like 1	-12	-11	-5	Other
PDPN	podoplanin	-12	-6	-9	Plasma Membrane
PXDC1	PX domain containing 1	-12	-10	-8	Other
PYGO1	pygopus family PHD finger 1	-12	-11	-9	Nucleus
KLRK1	killer cell lectin like receptor K1	-12	-9	-6	Plasma Membrane
HMCN1	hemicentin 1	-12	-13	-11	Extracellular Space
IKZF3	IKAROS family zinc finger 3	-13	-11	-7	Nucleus
TCIM	transcriptional and immune response regulator	-13	-11	-12	Nucleus
CEP85L	centrosomal protein 85 like	-13	-10	-11	Cytoplasm
CYTH3	cytohesin 3	-13	-11	-6	Cytoplasm
RP1	RP1 axonemal microtubule associated	-13	-10	-12	Cytoplasm
TBX21	T-box transcription factor 21	-13	-13	-12	Nucleus
PNLIPRP2	pancreatic lipase related protein 2 (gene/pseudogene)	-13	-18	-9	Extracellular Space
HERC6	HECT and RLD domain containing E3 ubiquitin protein ligase family member 6	-13	-8	-9	Cytoplasm
PDGFRA	platelet derived growth factor receptor alpha	-13	-18	-13	Plasma Membrane
HPGD	15-hydroxyprostaglandin dehydrogenase	-13	-43	-32	Cytoplasm
PTPRK	protein tyrosine phosphatase receptor type K	-14	-12	-14	Plasma Membrane
NEBL	nebulette	-14	-8	-9	Plasma Membrane
MMP8	matrix metallopeptidase 8	-14	-31	-27	Extracellular Space
TPPP3	tubulin polymerization promoting protein family member 3	-14	-5	-6	Cytoplasm
SEMA3G	semaphorin 3G	-14	-13	-11	Cytoplasm
IL2RB	interleukin 2 receptor subunit beta	-15	-13	-10	Plasma Membrane
Akr1cl	aldo-keto reductase family 1, member C-like	-15	-8	-7	Plasma Membrane
KLF4	KLF transcription factor 4	-15	-13	-8	Nucleus
ADGRE5	adhesion G protein-coupled receptor E5	-15	-23	-12	Plasma Membrane
Ddx60	DEXD/H-box helicase 60	-15	-14	-16	Cytoplasm
ITGA1	integrin subunit alpha 1	-15	-20	-16	Plasma Membrane
EFNB2	ephrin B2	-15	-40	-22	Plasma Membrane
PLSCR2	phospholipid scramblase 2	-15	-10	-13	Cytoplasm
GUCY1A1	guanylate cyclase 1 soluble subunit alpha 1	-16	-13	-9	Cytoplasm
TNFSF10	TNF superfamily member 10	-16	-6	-7	Extracellular Space
NEB	nebulin	-16	-16	-14	Cytoplasm
RAPGEF4	Rap guanine nucleotide exchange factor 4	-16	-17	-15	Cytoplasm
SCUBE2	signal peptide, CUB domain and EGF like domain containing 2	-17	-10	-7	Extracellular Space
BANK1	B cell scaffold protein with ankyrin repeats 1	-17	-11	-10	Extracellular Space
HYAL1	hyaluronidase 1	-17	-10	-7	Cytoplasm
CLDN5	claudin 5	-18	-33	-24	Plasma Membrane
ADGRL4	adhesion G protein-coupled receptor L4	-18	-38	-31	Plasma Membrane
DLK1	delta like non-canonical Notch ligand 1	-18	-21	-25	Extracellular Space
TIMP3	TIMP metallopeptidase inhibitor 3	-18	-15	-14	Extracellular Space
Klrb1f	killer cell lectin-like receptor subfamily B member 1F	-18	-12	-11	Plasma Membrane
PPP1R16B	protein phosphatase 1 regulatory subunit 16B	-18	-10	-6	Plasma Membrane
CLEC14A	C-type lectin domain containing 14A	-18	-6	-12	Cytoplasm
FOXF1	forkhead box F1	-18	-41	-28	Nucleus
OGN	osteoglycin	-18	-20	-6	Extracellular Space
MEIS1	Meis homeobox 1	-18	-21	-12	Nucleus
GIMAP4	GTPase, IMAP family member 4	-18	-16	-10	Nucleus
VIPR1	vasoactive intestinal peptide receptor 1	-18	-20	-9	Plasma Membrane
PDZD2	PDZ domain containing 2	-19	-9	-6	Plasma Membrane

Hbb-bs/Hbb-bt	hemoglobin, beta adult s chain	-19	-17	-19	Extracellular Space
GPR182	G protein-coupled receptor 182	-19	-24	-15	Plasma Membrane
CAVIN1	caveolae associated protein 1	-19	-18	-11	Nucleus
CYRIA	CYFIP related Rac1 interactor A	-19	-21	-17	Other
Gimap9	GTPase, IMAP family member 9	-19	-16	-14	Cytoplasm
ANKRD44	ankyrin repeat domain 44	-20	-13	-9	Other
CLIC5	chloride intracellular channel 5	-20	-7	-6	Cytoplasm
KL	klotho	-20	-21	-13	Extracellular Space
ABCA5	ATP binding cassette subfamily A member 5	-20	-9	-12	Plasma Membrane
TCF21	transcription factor 21	-20	-6	-4	Nucleus
ALDH1A1	aldehyde dehydrogenase 1 family member A1	-20	-9	-10	Cytoplasm
XPNPEP2	X-prolyl aminopeptidase 2	-20	-12	-7	Plasma Membrane
NKG7	natural killer cell granule protein 7	-20	-19	-16	Plasma Membrane
Cd300c2	CD300C molecule 2	-21	-14	-12	Plasma Membrane
CYP2E1	cytochrome P450 family 2 subfamily E member 1	-21	-32	-27	Cytoplasm
EDNRB	endothelin receptor type B	-21	-17	-8	Plasma Membrane
TMEM26	transmembrane protein 26	-21	-12	-13	Other
HEG1	heart development protein with EGF like domains 1	-21	-25	-12	Plasma Membrane
LIMCH1	LIM and calponin homology domains 1	-22	-12	-8	Cytoplasm
KLRD1	killer cell lectin like receptor D1	-22	-21	-8	Plasma Membrane
SLCO2A1	solute carrier organic anion transporter family member 2A1	-23	-9	-6	Plasma Membrane
Gvin1 (includes others)	GTPase, very large interferon inducible 1	-23	-18	-13	Nucleus
ACVRL1	activin A receptor like type 1	-24	-20	-9	Plasma Membrane
COL4A3	collagen type IV alpha 3 chain	-25	-15	-12	Extracellular Space
NPR3	natriuretic peptide receptor 3	-25	-82	-90	Plasma Membrane
Slfn3/Slfn4	schlafen family member 4	-26	-51	-74	Other
KLRC2	killer cell lectin like receptor C2	-26	-42	-22	Plasma Membrane
GPR15LG	G protein-coupled receptor 15 ligand	-26	-24	-17	Extracellular Space
THBD	thrombomodulin	-27	-22	-19	Plasma Membrane
Klrc2	killer cell lectin like receptor C2	-30	-28	-27	Plasma Membrane
Klrc2/Klrc3	killer cell lectin-like receptor subfamily C, member 2	-30	-20	-16	Plasma Membrane
FAR2	fatty acyl-CoA reductase 2	-30	-31	-28	Cytoplasm
Cdr1	cerebellar degeneration related antigen 1	-31	-22	-11	Other
CALCRL	calcitonin receptor like receptor	-31	-29	-15	Plasma Membrane
FMO1	flavin containing dimethylaniline monoxygenase 1	-31	-20	-6	Cytoplasm
Fmo3	flavin containing dimethylaniline monoxygenase 3	-32	-25	-17	Cytoplasm
SCN7A	sodium voltage-gated channel alpha subunit 7	-32	-33	-23	Plasma Membrane
GZMK	granzyme K	-36	-35	-28	Cytoplasm
CDO1	cysteine dioxygenase type 1	-37	-27	-22	Cytoplasm
CAVIN2	caveolae associated protein 2	-38	-19	-13	Plasma Membrane
PLVAP	plasmalemma vesicle associated protein	-41	-27	-15	Plasma Membrane
AKAP5	A-kinase anchoring protein 5	-42	-18	-19	Plasma Membrane
ADGRL3	adhesion G protein-coupled receptor L3	-42	-37	-21	Plasma Membrane
SYNE1	spectrin repeat containing nuclear envelope protein 1	-44	-46	-36	Nucleus
CLEC1A	C-type lectin domain family 1 member A	-44	-29	-29	Plasma Membrane
GLP1R	glucagon like peptide 1 receptor	-46	-25	-12	Plasma Membrane
CPED1	cadherin like and PC-esterase domain containing 1	-49	-25	-18	Cytoplasm
DUSP1	dual specificity phosphatase 1	-52	-14	-19	Nucleus
RTKN2	rhotekin 2	-55	-23	-21	Plasma Membrane
TEK	TEK receptor tyrosine kinase	-57	-67	-38	Plasma Membrane
CYYR1	cysteine and tyrosine rich 1	-58	-37	-17	Other
SEC14L3	SEC14 like lipid binding 3	-71	-27	-35	Other
NOSTRIN	nitric oxide synthase trafficking	-75	-32	-35	Cytoplasm
Gzmb/Gzmbl1	granzyme B	-86	-69	-39	Cytoplasm
Abcb1b	ATP-binding cassette, sub-family B member 1B	-87	-44	-43	Plasma Membrane
ACER2	alkaline ceramidase 2	-118	-68	-44	Cytoplasm
CCL5	C-C motif chemokine ligand 5	-124	-73	-41	Extracellular Space
LYVE1	lymphatic vessel endothelial hyaluronan receptor 1	-142	-68	-60	Plasma Membrane
KLRC1	killer cell lectin like receptor C1	-153	-113	-84	Plasma Membrane
TMEM100	transmembrane protein 100	-195	-101	-42	Plasma Membrane
GZMA	granzyme A	-196	-155	-134	Cytoplasm
FMO2	flavin containing dimethylaniline monoxygenase 2	-265	-108	-95	Cytoplasm
PRF1	perforin 1	-334	-96	-46	Cytoplasm

Supplementary Table S2

Table S2. DEGs Uniquely Identified in MWCNT-7-MM and Not Detected in Any ADCs.

Symbol	Entrez Gene Name	Fold changes (vs controls)	Location
MB	myoglobin	1231	Cytoplasm
ACTA1	actin alpha 1, skeletal muscle	1210	Cytoplasm
MYH1	myosin heavy chain 1	938	Plasma Membrane
TNN	tenascin N	921	Plasma Membrane
ASPN	asporin	901	Extracellular Space
COL14A1	collagen type XIV alpha 1 chain	852	Extracellular Space
TNFAIP6	TNF alpha induced protein 6	807	Extracellular Space
C1QTNF6	C1q and TNF related 6	693	Extracellular Space
MFAP5	microfibril associated protein 5	692	Extracellular Space

MYOT	myotilin	602	Cytoplasm
LOXL2	lysyl oxidase like 2	563	Nucleus
H19	H19 imprinted maternally expressed transcript	559	Other
LRRC15	leucine rich repeat containing 15	525	Plasma Membrane
MYH7	myosin heavy chain 7	501	Cytoplasm
XIRP2	xin actin binding repeat containing 2	423	Cytoplasm
MYBPC1	myosin binding protein C1	417	Cytoplasm
ATP2A1	ATPase sarcoplasmic/endoplasmic reticulum Ca2+ transporting 1	377	Cytoplasm
MYH2	myosin heavy chain 2	370	Cytoplasm
CREB3L1	cAMP responsive element binding protein 3 like 1	357	Nucleus
CDHR5	cadherin related family member 5	354	Plasma Membrane
Gm1096	--	299	Other
TTN	titin	291	Cytoplasm
DES	desmin	279	Cytoplasm
GPC1	glypican 1	257	Plasma Membrane
COL3A1	collagen type III alpha 1 chain	239	Extracellular Space
FBN1	fibrillin 1	238	Extracellular Space
COX6A2	cytochrome c oxidase subunit 6A2	231	Cytoplasm
TPM2	tropomyosin 2	230	Extracellular Space
ADGRD1	adhesion G protein-coupled receptor D1	224	Plasma Membrane
THBS4	thrombospondin 4	216	Extracellular Space
COL11A1	collagen type XI alpha 1 chain	201	Extracellular Space
FBLN2	fibulin 2	191	Extracellular Space
ANKRD23	ankyrin repeat domain 23	180	Nucleus
MYL11	myosin light chain 11	167	Cytoplasm
MMP2	matrix metallopeptidase 2	161	Extracellular Space
GALNT16	polypeptide N-acetylgalactosaminyltransferase 16	159	Cytoplasm
TNNT1	troponin T1, slow skeletal type	157	Cytoplasm
ADAMTS4	ADAM metallopeptidase with thrombospondin type 1 motif 4	152	Extracellular Space
AMPD1	adenosine monophosphate deaminase 1	149	Cytoplasm
MMP14	matrix metallopeptidase 14	148	Extracellular Space
Ct55	cancer/testis antigen 55	145	Cytoplasm
PRRX1	paired related homeobox 1	141	Nucleus
UCP1	uncoupling protein 1	141	Cytoplasm
ACTN2	actinin alpha 2	135	Nucleus
CD248	CD248 molecule	133	Plasma Membrane
KLHL41	kelch like family member 41	127	Cytoplasm
C10orf71	chromosome 10 open reading frame 71	126	Other
FSCN1	fascin actin-bundling protein 1	125	Cytoplasm
NDUFA4L2	NDUFA4 mitochondrial complex associated like 2	122	Cytoplasm
TRDN	triadin	120	Cytoplasm
PRAF2	PRA1 domain family member 2	119	Plasma Membrane
EPB41L3	erythrocyte membrane protein band 4.1 like 3	117	Plasma Membrane
RCN3	reticulocalbin 3	115	Cytoplasm
SH3PXD2B	SH3 and PX domains 2B	114	Cytoplasm
NRAP	nebulin related anchoring protein	114	Cytoplasm
SNAI2	snail family transcriptional repressor 2	113	Nucleus
ANGPTL2	angiopoietin like 2	104	Extracellular Space
MYL1	myosin light chain 1	103	Cytoplasm
COL5A2	collagen type V alpha 2 chain	100	Extracellular Space
CKM	creatine kinase, M-type	92	Cytoplasm
SULT1C3	sulfotransferase family 1C member 3	91	Cytoplasm
ACTN3	actinin alpha 3	90	Plasma Membrane
CCN4	cellular communication network factor 4	88	Extracellular Space
CTSK	cathepsin K	85	Cytoplasm
GLT8D2	glycosyltransferase 8 domain containing 2	84	Other
FKBP10	FKBP prolyl isomerase 10	84	Cytoplasm
CASQ1	calsequestrin 1	84	Cytoplasm
CCDC80	coiled-coil domain containing 80	83	Nucleus
AIF1L	allograft inflammatory factor 1 like	81	Plasma Membrane
TNNT3	troponin T3, fast skeletal type	81	Cytoplasm
ADAM12	ADAM metallopeptidase domain 12	80	Plasma Membrane
CASQ2	calsequestrin 2	80	Cytoplasm
CMYA5	cardiomyopathy associated 5	78	Cytoplasm
CLDN23	claudin 23	76	Plasma Membrane
EMILIN2	elastin microfibril interfacer 2	73	Extracellular Space
PYGM	glycogen phosphorylase, muscle associated	72	Cytoplasm
ALDH1L2	aldehyde dehydrogenase 1 family member L2	72	Cytoplasm
MYL3	myosin light chain 3	72	Cytoplasm
ASGR2	asialoglycoprotein receptor 2	71	Plasma Membrane
DCN	decorin	70	Extracellular Space
GABRA2	gamma-aminobutyric acid type A receptor subunit alpha2	68	Plasma Membrane
TNNC2	troponin C2, fast skeletal type	66	Cytoplasm
CRABP2	cellular retinoic acid binding protein 2	65	Cytoplasm
EBF1	EBF transcription factor 1	64	Nucleus
SRL	sarcalumenin	63	Cytoplasm
COL5A1	collagen type V alpha 1 chain	61	Extracellular Space
HJV	hemojuvelin BMP co-receptor	60	Plasma Membrane
COL6A1	collagen type VI alpha 1 chain	59	Extracellular Space
COPZ2	COPI coat complex subunit zeta 2	54	Cytoplasm
ITIH5	inter-alpha-trypsin inhibitor heavy chain 5	54	Plasma Membrane
Prss32	serine protease 32	51	Extracellular Space
LRRC17	leucine rich repeat containing 17	50	Extracellular Space
ZNF469	zinc finger protein 469	49	Nucleus
FITM1	fat storage inducing transmembrane protein 1	49	Extracellular Space
TPM3	tropomyosin 3	47	Cytoplasm
FAP	fibroblast activation protein alpha	47	Cytoplasm
NID1	nidogen 1	47	Extracellular Space
IGSF3	immunoglobulin superfamily member 3	45	Plasma Membrane
HSPG2	heparan sulfate proteoglycan 2	45	Extracellular Space
COL6A2	collagen type VI alpha 2 chain	44	Extracellular Space
SLN	sarcolipin	43	Cytoplasm
LMOD2	leiomodin 2	43	Other
PRRX2	paired related homeobox 2	43	Nucleus
CA6	carbonic anhydrase 6	43	Extracellular Space
COL8A1	collagen type VIII alpha 1 chain	42	Extracellular Space

LCTL	lactase like	42	Cytoplasm
Nxf7	nuclear RNA export factor 7	41	Cytoplasm
NKAIN1	sodium/potassium transporting ATPase interacting 1	41	Plasma Membrane
CD34	CD34 molecule	40	Plasma Membrane
Samt2	spermatogenesis associated multipass transmembrane protein 2	40	Other
TCAP	titin-cap	40	Cytoplasm
PROKR2	prokineticin receptor 2	39	Plasma Membrane
ADAMTS5	ADAM metallopeptidase with thrombospondin type 1 motif 5	39	Extracellular Space
FMR1NB	FMR1 neighbor	39	Nucleus
Lcn4l3/Lcnl	Lipocalin like	37	Other
DLX3	distal-less homeobox 3	37	Nucleus
FKBP9	FKBP prolyl isomerase 9	37	Cytoplasm
DDR2	discoidin domain receptor tyrosine kinase 2	34	Plasma Membrane
1700013H16Rik	RIKEN cDNA 1700013H16 gene	34	Nucleus
TXLNB	taxilin beta	34	Cytoplasm
ANGPT4	angiopoietin 4	33	Extracellular Space
HTR2A	5-hydroxytryptamine receptor 2A	33	Plasma Membrane
LAMB1	laminin subunit beta 1	33	Extracellular Space
TMC3	transmembrane channel like 3	33	Other
RYR1	ryanodine receptor 1	33	Cytoplasm
HTRA3	HtrA serine peptidase 3	33	Extracellular Space
LSMEM1	leucine rich single-pass membrane protein 1	32	Cytoplasm
ENO3	enolase 3	32	Cytoplasm
NRK	Nik related kinase	32	Other
GXYLT2	glucoside xylosyltransferase 2	32	Other
TEX101	testis expressed 101	32	Plasma Membrane
TIMP1	TIMP metallopeptidase inhibitor 1	31	Extracellular Space
MYOZ2	myozenin 2	31	Cytoplasm
SCARA5	scavenger receptor class A member 5	30	Plasma Membrane
IRS1	insulin receptor substrate 1	30	Cytoplasm
PDGFC	platelet derived growth factor C	30	Extracellular Space
SSC5D	scavenger receptor cysteine rich family member with 5 domains	30	Plasma Membrane
LMOD3	leiomodin 3	29	Cytoplasm
ITGB7	integrin subunit beta 7	29	Plasma Membrane
CAVIN3	caveolae associated protein 3	28	Cytoplasm
COL12A1	collagen type XII alpha 1 chain	28	Extracellular Space
ALDH18A1	aldehyde dehydrogenase 18 family member A1	28	Cytoplasm
ADAMTS2	ADAM metallopeptidase with thrombospondin type 1 motif 2	27	Extracellular Space
TIMP4	TIMP metallopeptidase inhibitor 4	27	Extracellular Space
PDZRN3	PDZ domain containing ring finger 3	27	Extracellular Space
LOXL1	lysyl oxidase like 1	27	Extracellular Space
TUBB2B	tubulin beta 2B class IIb	27	Plasma Membrane
MMP23B	matrix metallopeptidase 23B	26	Extracellular Space
CCN3	cellular communication network factor 3	26	Extracellular Space
FSTL1	follistatin like 1	25	Extracellular Space
PRSS12	serine protease 12	25	Extracellular Space
SCN4A	sodium voltage-gated channel alpha subunit 4	25	Plasma Membrane
OSR1	odd-skipped related transcription factor 1	25	Nucleus
KREMEN1	kringle containing transmembrane protein 1	25	Plasma Membrane
C1S	complement C1s	24	Extracellular Space
SMOC2	SPARC related modular calcium binding 2	24	Extracellular Space
Zfp950l4	zinc finger protein 950 like 4	24	Other
TEX11	testis expressed 11	24	Nucleus
MYOM1	myomesin 1	24	Cytoplasm
Ptprv	protein tyrosine phosphatase receptor type V	24	Plasma Membrane
CACNA2D1	calcium voltage-gated channel auxiliary subunit alpha2delta 1	23	Plasma Membrane
PLOD2	procollagen-lysine,2-oxoglutarate 5-dioxygenase 2	23	Cytoplasm
CERCAM	cerebral endothelial cell adhesion molecule	23	Other
NAV3	neuron navigator 3	23	Nucleus
KLK1	kallikrein 1	23	Nucleus
NRCAM	neuronal cell adhesion molecule	23	Plasma Membrane
ARG1	arginase 1	23	Cytoplasm
P4HA3	prolyl 4-hydroxylase subunit alpha 3	22	Cytoplasm
MATN2	matrilin 2	22	Extracellular Space
SERPINH1	serpin family H member 1	22	Extracellular Space
PIWIL4	piwi like RNA-mediated gene silencing 4	22	Cytoplasm
GAA	alpha glucosidase	21	Cytoplasm
SCARA3	scavenger receptor class A member 3	21	Plasma Membrane
TGFB3	transforming growth factor beta 3	21	Extracellular Space
EN1	engrailed homeobox 1	21	Nucleus
ARSJ	arylsulfatase family member J	20	Extracellular Space
CPZ	carboxypeptidase Z	20	Extracellular Space
PDE4DIP	phosphodiesterase 4D interacting protein	20	Cytoplasm
IRAK3	interleukin 1 receptor associated kinase 3	20	Cytoplasm
MYH3	myosin heavy chain 3	20	Cytoplasm
MYH4	myosin heavy chain 4	20	Cytoplasm
CAPN6	calpain 6	20	Cytoplasm
SMO	smoothened, frizzled class receptor	19	Plasma Membrane
SMYD1	SET and MYND domain containing 1	19	Nucleus
DNM1	dynamin 1	19	Cytoplasm
ZDBF2	zinc finger DBF-type containing 2	19	Nucleus
DPYSL3	dihydropyrimidinase like 3	19	Cytoplasm
Znf431l12	zinc finger protein 431 like 12	19	Other
TRIM54	tripartite motif containing 54	19	Cytoplasm
PADI2	peptidyl arginine deiminase 2	19	Cytoplasm
ITGBL1	integrin subunit beta like 1	19	Extracellular Space
IGFBP6	insulin like growth factor binding protein 6	19	Extracellular Space
CRABP1	cellular retinoic acid binding protein 1	19	Cytoplasm
MYOM2	myomesin 2	19	Cytoplasm
COL5A3	collagen type V alpha 3 chain	18	Extracellular Space
Vwa5al1	von Willebrand factor A domain containing 5A like 1	18	Other
MYPN	myopalladin	18	Cytoplasm
Erhl1	ERH, mRNA splicing and mitosis factor like 1	18	Other
CIDEC	cell death inducing DFFA like effector c	18	Cytoplasm
PYCR1	pyrroline-5-carboxylate reductase 1	18	Cytoplasm
TNFSF9	TNF superfamily member 9	18	Plasma Membrane

MMP19	matrix metallopeptidase 19	18	Extracellular Space
SATB2	SATB homeobox 2	17	Nucleus
USP13	ubiquitin specific peptidase 13	17	Cytoplasm
MYBPC2	myosin binding protein C2	17	Cytoplasm
TLN2	talin 2	17	Nucleus
RPL10L	ribosomal protein L10 like	17	Nucleus
USP26	ubiquitin specific peptidase 26	17	Cytoplasm
CAVIN4	caveolae associated protein 4	17	Cytoplasm
CDH6	cadherin 6	17	Plasma Membrane
TNFSF18	TNF superfamily member 18	16	Extracellular Space
KCND1	potassium voltage-gated channel subfamily D member 1	16	Plasma Membrane
CHRNA1	cholinergic receptor nicotinic alpha 1 subunit	16	Plasma Membrane
ILDR2	immunoglobulin like domain containing receptor 2	16	Plasma Membrane
TRIM72	tripartite motif containing 72	16	Cytoplasm
SEC16B	SEC16 homolog B, endoplasmic reticulum export factor	16	Nucleus
NES	nestin	16	Extracellular Space
SCML2	Scm polycomb group protein like 2	16	Nucleus
HSPB6	heat shock protein family B (small) member 6	16	Cytoplasm
CACNA1S	calcium voltage-gated channel subunit alpha1 S	16	Plasma Membrane
STEAP1	STEAP family member 1	16	Plasma Membrane
AFAP1	actin filament associated protein 1	15	Cytoplasm
FLNC	filamin C	15	Cytoplasm
3830403N18Rik/Xlr	X-linked lymphocyte-regulated	15	Nucleus
TMEM182	transmembrane protein 182	15	Other
CREB5	cAMP responsive element binding protein 5	15	Nucleus
GALNT17	polypeptide N-acetylgalactosaminyltransferase 17	15	Cytoplasm
OLFML3	olfactomedin like 3	15	Extracellular Space
SPATS2L	spermatogenesis associated serine rich 2 like	15	Nucleus
TUBB3	tubulin beta 3 class III	15	Cytoplasm
MARCKSL1	MARCKS like 1	15	Cytoplasm
CAP2	cyclase associated actin cytoskeleton regulatory protein 2	14	Plasma Membrane
STMN4	stathmin 4	14	Cytoplasm
TRIM29	tripartite motif containing 29	14	Cytoplasm
TNMD	tenomodulin	14	Plasma Membrane
PPP1R3A	protein phosphatase 1 regulatory subunit 3A	14	Cytoplasm
SPP2	secreted phosphoprotein 2	14	Extracellular Space
Folr2	folate receptor beta	14	Plasma Membrane
SCN1B	sodium voltage-gated channel beta subunit 1	14	Plasma Membrane
SNCG	synuclein gamma	14	Cytoplasm
CSRP3	cysteine and glycine rich protein 3	14	Nucleus
LIMK1	LIM domain kinase 1	13	Cytoplasm
VASH1	vasohibin 1	13	Extracellular Space
ZNF75D	zinc finger protein 75D	13	Other
ALDH8A1	aldehyde dehydrogenase 8 family member A1	13	Cytoplasm
ANO5	anoctamin 5	13	Plasma Membrane
RAB34	RAB34, member RAS oncogene family	13	Cytoplasm
CGREF1	cell growth regulator with EF-hand domain 1	13	Extracellular Space
C15orf62	chromosome 15 open reading frame 62	13	Cytoplasm
APOD	apolipoprotein D	13	Extracellular Space
CPT1B	carnitine palmitoyltransferase 1B	13	Cytoplasm
CDR2L	cerebellar degeneration related protein 2 like	13	Other
PAPPA	pappalysin 1	13	Extracellular Space
PDLIM7	PDZ and LIM domain 7	13	Cytoplasm
C1QTNF1	C1q and TNF related 1	12	Extracellular Space
DBN1	drebrin 1	12	Cytoplasm
SPINK2	serine peptidase inhibitor Kazal type 2	12	Extracellular Space
CAPN3	calpain 3	12	Cytoplasm
P3H1	prolyl 3-hydroxylase 1	12	Nucleus
TFPI2	tissue factor pathway inhibitor 2	12	Extracellular Space
SELENOM	selenoprotein M	12	Cytoplasm
SLC41A2	solute carrier family 41 member 2	12	Plasma Membrane
CALHM2	calcium homeostasis modulator family member 2	12	Plasma Membrane
PEG10	paternally expressed 10	12	Nucleus
SCRN1	secernin 1	11	Cytoplasm
Rhox9/Rhox9l1	reproductive homeobox 9	11	Other
MYL4	myosin light chain 4	11	Cytoplasm
Zfp882	zinc finger protein 882	11	Other
P3H4	prolyl 3-hydroxylase family member 4 (inactive)	11	Nucleus
MLIP	muscular LMNA interacting protein	11	Nucleus
PVALB	parvalbumin	11	Cytoplasm
PTK7	protein tyrosine kinase 7 (inactive)	11	Plasma Membrane
PTPRQ	protein tyrosine phosphatase receptor type Q	11	Extracellular Space
CCDC8	coiled-coil domain containing 8	11	Plasma Membrane
REDIC1	regulator of DNA class I crossover intermediates 1	11	Other
SGCG	sarcoglycan gamma	11	Plasma Membrane
HHIPL1	HHIP like 1	11	Other
TRPM8	transient receptor potential cation channel subfamily M member 8	11	Plasma Membrane
FN1	fibronectin 1	10	Extracellular Space
SLC9B1	solute carrier family 9 member B1	10	Plasma Membrane
Xlr4a	X-linked lymphocyte-regulated 4A	10	Other
ANKRD2	ankyrin repeat domain 2	10	Nucleus
HSPB7	heat shock protein family B (small) member 7	10	Cytoplasm
PLPPR5	phospholipid phosphatase related 5	9	Plasma Membrane
OR4C3	olfactory receptor family 4 subfamily C member 3	9	Plasma Membrane
NTMT2	N-terminal Xaa-Pro-Lys N-methyltransferase 2	9	Nucleus
Gm773	predicted gene 773	9	Nucleus
LOC100911047/Xlr3a	X-linked lymphocyte-regulated 3A	9	Other
SLC41A3	solute carrier family 41 member 3	9	Plasma Membrane
PCK1	phosphoenolpyruvate carboxykinase 1	9	Cytoplasm
MYO7A	myosin VIIA	9	Cytoplasm
COX7B2	cytochrome c oxidase subunit 7B2	9	Cytoplasm
PIWIL2	piwi like RNA-mediated gene silencing 2	8	Cytoplasm
TMEM38A	transmembrane protein 38A	8	Cytoplasm
SMTNL1	smoothelin like 1	8	Cytoplasm
Defb37	defensin beta 37	8	Other
PCDHB2	protocadherin beta 2	8	Plasma Membrane

LRRN4CL	LRRN4 C-terminal like	8	Other
CHRND	cholinergic receptor nicotinic delta subunit	8	Plasma Membrane
PAK3	p21 (RAC1) activated kinase 3	8	Cytoplasm
RTL9	retrotransposon Gag like 9	8	Other
PDE10A	phosphodiesterase 10A	8	Cytoplasm
TDRD9	tudor domain containing 9	8	Cytoplasm
CCNB3	cyclin B3	7	Nucleus
BMAL2	basic helix-loop-helix ARNT like 2	7	Nucleus
TENM3	teneurin transmembrane protein 3	7	Plasma Membrane
LRRC75B	leucine rich repeat containing 75B	7	Other
GRIN2A	glutamate ionotropic receptor NMDA type subunit 2A	7	Plasma Membrane
TNNI2	troponin I2, fast skeletal type	7	Cytoplasm
SLC26A5	solute carrier family 26 member 5	7	Plasma Membrane
PCDHB3	protocadherin beta 3	7	Other
ART3	ADP-ribosyltransferase 3 (inactive)	7	Plasma Membrane
KCNH1	potassium voltage-gated channel subfamily H member 1	7	Plasma Membrane
TCEAL7	transcription elongation factor A like 7	7	Nucleus
LDB3	LIM domain binding 3	7	Cytoplasm
SFMBT2	Scm like with four mbt domains 2	7	Nucleus
CAMK2B	calcium/calmodulin dependent protein kinase II beta	7	Cytoplasm
ANO7	anoctamin 7	7	Plasma Membrane
AA414768	expressed sequence AA414768	7	Other
NEFL	neurofilament light chain	6	Cytoplasm
2310002L09Rik	RIKEN cDNA 2310002L09 gene	6	Cytoplasm
XIRP1	xin actin binding repeat containing 1	6	Plasma Membrane
CSMD3	CUB and Sushi multiple domains 3	6	Plasma Membrane
SV2C	synaptic vesicle glycoprotein 2C	6	Plasma Membrane
KCNG4	potassium voltage-gated channel modifier subfamily G member 4	6	Plasma Membrane
STMN2	stathmin 2	6	Plasma Membrane
CACNG6	calcium voltage-gated channel auxiliary subunit gamma 6	6	Plasma Membrane
TBX18	T-box transcription factor 18	6	Nucleus
Igbp1b	immunoglobulin (CD79A) binding protein 1b	6	Cytoplasm
GREM1	gremlin 1, DAN family BMP antagonist	6	Extracellular Space
SPARC	secreted protein acidic and cysteine rich	6	Extracellular Space
SLC38A4	solute carrier family 38 member 4	5	Plasma Membrane
FEZ1	fasciculation and elongation protein zeta 1	5	Cytoplasm
MYOZ1	myozenin 1	5	Cytoplasm
DYNC1I1	dynein cytoplasmic 1 intermediate chain 1	5	Cytoplasm
LRRC2	leucine rich repeat containing 2	5	Other
SOX11	SRY-box transcription factor 11	5	Nucleus
ASB15	ankyrin repeat and SOCS box containing 15	5	Nucleus
SPSB2	splA/ryanodine receptor domain and SOCS box containing 2	5	Cytoplasm
RRAD	RRAD, Ras related glycolysis inhibitor and calcium channel regulator	5	Cytoplasm
SLC6A17	solute carrier family 6 member 17	5	Cytoplasm
SYNPO2L	synaptopodin 2 like	5	Cytoplasm
TKTL1	transketolase like 1	5	Cytoplasm
KLHL34	kelch like family member 34	5	Extracellular Space
SHC2	SHC adaptor protein 2	5	Cytoplasm
LHX2	LIM homeobox 2	5	Nucleus
MAP1A	microtubule associated protein 1A	5	Cytoplasm
MYOG	myogenin	5	Nucleus
Serpinb3a	serine (or cysteine) peptidase inhibitor, clade B (ovalbumin), member 3A	5	Cytoplasm
HOXB7	homeobox B7	5	Nucleus
MYLK4	myosin light chain kinase family member 4	5	Cytoplasm
BFSP1	beaded filament structural protein 1	5	Cytoplasm
EXO1	exonuclease 1	5	Nucleus
Mmp3	matrix metallopeptidase 3	5	Extracellular Space
ABLIM2	actin binding LIM protein family member 2	5	Cytoplasm
RAB3IL1	RAB3A interacting protein like 1	5	Cytoplasm
CACNA1I	calcium voltage-gated channel subunit alpha1 I	5	Plasma Membrane
SLC36A2	solute carrier family 36 member 2	5	Plasma Membrane
TCEANC	transcription elongation factor A N-terminal and central domain containing	5	Other
B3GNT9	UDP-GlcNAc:betaGal beta-1,3-N-acetylglucosaminyltransferase 9	5	Cytoplasm
MLANA	melan-A	5	Plasma Membrane
SEPTIN5	septin 5	5	Cytoplasm
PDLIM4	PDZ and LIM domain 4	5	Plasma Membrane
RGD1565323	similar to OTTMUSP00000000621	5	Other
Zscan4c (includes others)	zinc finger and SCAN domain containing 4C	5	Nucleus
P2RX5	purinergic receptor P2X 5	5	Plasma Membrane
Myh8	myosin heavy chain 8	4	Cytoplasm
LGI4	leucine rich repeat LGI family member 4	4	Extracellular Space
FBLN7	fibulin 7	4	Extracellular Space
EFCAB3	EF-hand calcium binding domain 3	4	Other
GARIN1B	golgi associated RAB2 interactor 1B	4	Cytoplasm
CACNG1	calcium voltage-gated channel auxiliary subunit gamma 1	4	Plasma Membrane
FSD1	fibronectin type III and SPRY domain containing 1	4	Cytoplasm
AASS	aminoadipate-semialdehyde synthase	4	Cytoplasm
ABRA	actin binding Rho activating protein	4	Cytoplasm
CDNF	cerebral dopamine neurotrophic factor	4	Other
DDIAS	DNA damage induced apoptosis suppressor	4	Cytoplasm
Cyp2c29 (includes others)	cytochrome P450, family 2, subfamily c, polypeptide 29	4	Cytoplasm
FKBP14	FKBP prolyl isomerase 14	4	Cytoplasm
GRID1	glutamate ionotropic receptor delta type subunit 1	4	Plasma Membrane
NPY1R	neuropeptide Y receptor Y1	4	Plasma Membrane
PIANP	PILR alpha associated neural protein	4	Plasma Membrane
MYH6	myosin heavy chain 6	4	Cytoplasm
ALPK3	alpha kinase 3	4	Nucleus
OBSCN	obscurin, cytoskeletal calmodulin and titin-interacting RhoGEF	4	Cytoplasm
Mgst3l1	microsomal glutathione S-transferase 3 like 1	4	Other
FHL3	four and a half LIM domains 3	4	Plasma Membrane
METTL1	methyltransferase 1, tRNA methylguanosine	4	Nucleus
FOXS1	forkhead box S1	4	Nucleus
ZBTB8A	zinc finger and BTB domain containing 8A	4	Nucleus
RAB11FIP5	RAB11 family interacting protein 5	4	Cytoplasm
ISLR2	immunoglobulin superfamily containing leucine rich repeat 2	4	Plasma Membrane
NECAB1	N-terminal EF-hand calcium binding protein 1	4	Cytoplasm

C1QTNF3	C1q and TNF related 3	4	Extracellular Space
PPP3R2	protein phosphatase 3 regulatory subunit B, beta	4	Cytoplasm
SYPL2	synaptophysin like 2	4	Other
PLIN1	perilipin 1	4	Cytoplasm
TRIM55	tripartite motif containing 55	4	Cytoplasm
MAB21L3	mab-21 like 3	4	Other
GNG11	G protein subunit gamma 11	4	Plasma Membrane
COL7A1	collagen type VII alpha 1 chain	4	Extracellular Space
MATCAP1	microtubule associated tyrosine carboxypeptidase 1	4	Cytoplasm
TRMT61A	tRNA methyltransferase 61A	4	Nucleus
PPP1R27	protein phosphatase 1 regulatory subunit 27	4	Nucleus
AKAP6	A-kinase anchoring protein 6	4	Nucleus
SMYD5	SMYD family member 5	4	Nucleus
RNF113A	ring finger protein 113A	4	Nucleus
SIX1	SIX homeobox 1	4	Nucleus
SHOX2	SHOX homeobox 2	4	Nucleus
IGF2BP3	insulin like growth factor 2 mRNA binding protein 3	4	Cytoplasm
CPXM1	carboxypeptidase X, M14 family member 1	4	Extracellular Space
Ptgdrl1	prostaglandin D2 receptor-like 1	4	Other
GPSM2	G protein signaling modulator 2	4	Nucleus
SYCE2	synaptonemal complex central element protein 2	4	Nucleus
NHSL1	NHS like 1	4	Other
MICALL2	MICAL like 2	4	Cytoplasm
RXRG	retinoid X receptor gamma	4	Nucleus
E2F7	E2F transcription factor 7	4	Nucleus
mir-199 (includes others)	relatives of microRNA 199	4	Cytoplasm
Pcdhb22	protocadherin beta 22	4	Extracellular Space
BEST3	bestrophin 3	4	Nucleus
PM20D1	peptidase M20 domain containing 1	4	Cytoplasm
ITGA7	integrin subunit alpha 7	3	Plasma Membrane
SLC38A11	solute carrier family 38 member 11	3	Other
ADAMTSL5	ADAMTS like 5	3	Extracellular Space
PGAM2	phosphoglycerate mutase 2	3	Cytoplasm
Pgk1l1	phosphoglycerate kinase 1 like 1	3	Other
LDOC1	LDOC1 regulator of NFKB signaling	3	Nucleus
TRIM63	tripartite motif containing 63	3	Nucleus
SPIN4	spindlin family member 4	3	Nucleus
MYL2	myosin light chain 2	3	Cytoplasm
EEF1A2	eukaryotic translation elongation factor 1 alpha 2	3	Cytoplasm
CRYBG1	crystallin beta-gamma domain containing 1	-3	Extracellular Space
MAL	mal, T cell differentiation protein	-3	Plasma Membrane
LY75	lymphocyte antigen 75	-3	Plasma Membrane
TRAF3IP3	TRAF3 interacting protein 3	-3	Cytoplasm
LRG1	leucine rich alpha-2-glycoprotein 1	-3	Extracellular Space
MARVELD2	MARVEL domain containing 2	-4	Plasma Membrane
RHOH	ras homolog family member H	-4	Plasma Membrane
CPVL	carboxypeptidase vitellogenic like	-4	Cytoplasm
TAFA3	TAFA chemokine like family member 3	-4	Extracellular Space
TMEM184A	transmembrane protein 184A	-4	Cytoplasm
Atp5mgl4	ATP synthase membrane subunit G like 4	-4	Other
MBNL3	muscleblind like splicing regulator 3	-4	Nucleus
SEMA4F	ssemaphorin 4F	-4	Plasma Membrane
EREG	epiregulin	-4	Extracellular Space
TSTD1	thiosulfate sulfurtransferase like domain containing 1	-4	Cytoplasm
IRX2	iroquois homeobox 2	-4	Nucleus
FRAS1	Fraser extracellular matrix complex subunit 1	-4	Extracellular Space
SLC24A4	solute carrier family 24 member 4	-4	Plasma Membrane
AGBL2	AGBL carboxypeptidase 2	-4	Cytoplasm
PRDM1	PR/SET domain 1	-4	Nucleus
RSPH10B	radial spoke head 10 homolog B	-4	Extracellular Space
Sirpb2l1	signal-regulatory protein beta 2-like 1	-4	Other
CMTM8	CKLF like MARVEL transmembrane domain containing 8	-4	Extracellular Space
SLC6A20	solute carrier family 6 member 20	-4	Plasma Membrane
ATP13A4	ATPase 13A4	-4	Cytoplasm
STX3	syntaxin 3	-4	Plasma Membrane
CD96	CD96 molecule	-4	Plasma Membrane
RBPJL	recombination signal binding protein for immunoglobulin kappa J region like	-4	Nucleus
HOOK1	hook microtubule tethering protein 1	-4	Cytoplasm
Igkvl15	immunoglobulin kappa variable like 15	-4	Other
GRAMD1C	GRAM domain containing 1C	-4	Cytoplasm
SLC44A4	solute carrier family 44 member 4	-4	Plasma Membrane
NEXMIF	neurite extension and migration factor	-4	Nucleus
EPN3	epsin 3	-4	Cytoplasm
CXCR6	C-X-C motif chemokine receptor 6	-4	Plasma Membrane
SLC1A3	solute carrier family 1 member 3	-4	Plasma Membrane
HP	haptoglobin	-4	Extracellular Space
CCSER1	coiled-coil serine rich protein 1	-4	Other
ZSWIM5	zinc finger SWIM-type containing 5	-4	Extracellular Space
SLC37A1	solute carrier family 37 member 1	-4	Plasma Membrane
TTC6	tetratricopeptide repeat domain 6	-4	Other
BSPRY	B-box and SPRY domain containing	-4	Cytoplasm
GIMAP1	GTPase, IMAP family member 1	-4	Cytoplasm
CYB561	cytochrome b561	-4	Cytoplasm
Snhg11	small nucleolar RNA host gene 11	-4	Other
KCNAB2	potassium voltage-gated channel subfamily A regulatory beta subunit 2	-5	Plasma Membrane
DENND2D	DENN domain containing 2D	-5	Cytoplasm
Cyp3a13	cytochrome P450, family 3, subfamily a, polypeptide 13	-5	Cytoplasm
RASEF	RAS and EF-hand domain containing	-5	Cytoplasm
IRX1	iroquois homeobox 1	-5	Nucleus
LOC102553828	spermatogenesis-associated protein 13-like	-5	Other
Zfp758l1	zinc finger protein 758 like 1	-5	Other
UNC13D	unc-13 homolog D	-5	Cytoplasm
CCDC146	coiled-coil domain containing 146	-5	Cytoplasm
PLCH1	phospholipase C eta 1	-5	Cytoplasm
CSF3R	colony stimulating factor 3 receptor	-5	Plasma Membrane
ST6GALNAC3	ST6 N-acetylgalactosaminide alpha-2,6-sialyltransferase 3	-5	Cytoplasm

RSPH4A	radial spoke head component 4A	-5	Nucleus
CACNA1D	calcium voltage-gated channel subunit alpha1 D	-5	Plasma Membrane
CPN1	carboxypeptidase N subunit 1	-5	Extracellular Space
UPK1B	uroplakin 1B	-5	Plasma Membrane
ARHGEF38	Rho guanine nucleotide exchange factor 38	-5	Other
DNAH11	dynein axonemal heavy chain 11	-5	Cytoplasm
PDE9A	phosphodiesterase 9A	-5	Cytoplasm
MYB	MYB proto-oncogene, transcription factor	-6	Nucleus
SPAG17	sperm associated antigen 17	-6	Cytoplasm
Nat8f4	N-acetyltransferase 8 (GCN5-related) family member 4	-6	Cytoplasm
Lilrc2	leukocyte immunoglobulin-like receptor, subfamily C, member 2	-6	Other
CLXN	calaxin	-6	Cytoplasm
MMD	monocyte to macrophage differentiation associated	-6	Plasma Membrane
PCSK1	proprotein convertase subtilisin/kexin type 1	-7	Cytoplasm
Gm6377	predicted gene 6377	-7	Other
TOX3	TOX high mobility group box family member 3	-8	Nucleus
VSIG4	V-set and immunoglobulin domain containing 4	-8	Plasma Membrane
CAPSL	calcyphosine like	-9	Other
RAB27B	RAB27B, member RAS oncogene family	-9	Cytoplasm
ZFP36	ZFP36 ring finger protein	-10	Nucleus
INPP1	inositol polyphosphate-1-phosphatase	-10	Cytoplasm
Cd177	CD177 antigen	-10	Other
Pilrb1/Pilrb2	paired immunoglobin-like type 2 receptor beta 2	-10	Plasma Membrane
SHROOM4	shroom family member 4	-11	Plasma Membrane
KCNA3	potassium voltage-gated channel subfamily A member 3	-11	Plasma Membrane
LRATD2	LRAT domain containing 2	-11	Plasma Membrane
SCIMP	SLP adaptor and CSK interacting membrane protein	-11	Plasma Membrane
SLC1A1	solute carrier family 1 member 1	-11	Plasma Membrane
KLF5	KLF transcription factor 5	-11	Nucleus
ENPP4	ectonucleotide pyrophosphatase/phosphodiesterase 4	-11	Cytoplasm
IGSF5	immunoglobulin superfamily member 5	-12	Plasma Membrane
PLA2G7	phospholipase A2 group VII	-12	Extracellular Space
Ctla2a	cytotoxic T lymphocyte-associated protein 2 alpha	-12	Other
CADPS2	calcium dependent secretion activator 2	-12	Plasma Membrane
ANK3	ankyrin 3	-12	Plasma Membrane
KCNJ15	potassium inwardly rectifying channel subfamily J member 15	-12	Plasma Membrane
EPS8L1	EPS8 signaling adaptor L1	-12	Cytoplasm
IER2	immediate early response 2	-12	Cytoplasm
SQOR	sulfide quinone oxidoreductase	-12	Cytoplasm
IFIH1	interferon induced with helicase C domain 1	-12	Nucleus
IFI44L	interferon induced protein 44 like	-12	Nucleus
SYDE2	synapse defective Rho GTPase homolog 2	-13	Cytoplasm
ABCA6	ATP binding cassette subfamily A member 6	-13	Plasma Membrane
C1orf116	chromosome 1 open reading frame 116	-13	Cytoplasm
REPS2	RALBP1 associated Eps domain containing 2	-13	Cytoplasm
MCTP2	multiple C2 and transmembrane domain containing 2	-13	Other
PIK3CB	phosphatidylinositol-4,5-bisphosphate 3-kinase catalytic subunit beta	-13	Cytoplasm
ERBB3	erb-b2 receptor tyrosine kinase 3	-13	Plasma Membrane
SH2D4A	SH2 domain containing 4A	-13	Cytoplasm
IL1B	interleukin 1 beta	-14	Extracellular Space
CXCR4	C-X-C motif chemokine receptor 4	-14	Plasma Membrane
MYO1G	myosin IG	-14	Cytoplasm
RASGRF2	Ras protein specific guanine nucleotide releasing factor 2	-14	Cytoplasm
WFDC2	WAP four-disulfide core domain 2	-14	Extracellular Space
TMEM243	transmembrane protein 243	-14	Other
ZDHHC2	zinc finger DHHC-type palmitoyltransferase 2	-14	Nucleus
SPINT2	serine peptidase inhibitor, Kunitz type 2	-14	Extracellular Space
FOXA1	forkhead box A1	-15	Nucleus
FGFR3	fibroblast growth factor receptor 3	-15	Plasma Membrane
CCDC141	coiled-coil domain containing 141	-15	Cytoplasm
MECOM	MDS1 and EVI1 complex locus	-15	Nucleus
Igh-6	immunoglobulin heavy chain 6	-15	Other
MOXD1	monooxygenase DBH like 1	-16	Cytoplasm
CADM1	cell adhesion molecule 1	-16	Plasma Membrane
Cyfip2	cytoplasmic FMR1 interacting protein 2	-16	Cytoplasm
FLVCR2	FLVCR choline and putative heme transporter 2	-16	Plasma Membrane
Ccl3	C-C motif chemokine ligand 3	-17	Extracellular Space
PPL	periplakin	-17	Cytoplasm
C5	complement C5	-18	Extracellular Space
SPINT1	serine peptidase inhibitor, Kunitz type 1	-18	Extracellular Space
NECTIN3	nectin cell adhesion molecule 3	-18	Plasma Membrane
DAPK1	death associated protein kinase 1	-18	Cytoplasm
Lyz1/Lyz2	lysozyme 2	-18	Cytoplasm
GNA14	G protein subunit alpha 14	-18	Plasma Membrane
NKD1	NKD inhibitor of WNT signaling pathway 1	-19	Other
BCAM	basal cell adhesion molecule (Lutheran blood group)	-19	Plasma Membrane
SORT1	sortilin 1	-19	Plasma Membrane
CPM	carboxypeptidase M	-19	Plasma Membrane
NCKAP5	NCK associated protein 5	-20	Other
Lilrb4a/Lilrb4b	leukocyte immunoglobulin-like receptor, subfamily B, member 4A	-20	Plasma Membrane
SELENBP1	selenium binding protein 1	-21	Cytoplasm
RAB11FIP1	RAB11 family interacting protein 1	-21	Cytoplasm
EMB	embigin	-21	Plasma Membrane
LAMP3	lysosomal associated membrane protein 3	-21	Plasma Membrane
NAV2	neuron navigator 2	-22	Nucleus
ID2	inhibitor of DNA binding 2	-22	Nucleus
AFAP1L1	actin filament associated protein 1 like 1	-22	Other
PTPRC	protein tyrosine phosphatase receptor type C	-22	Plasma Membrane
MGST1	microsomal glutathione S-transferase 1	-23	Cytoplasm
EPB41L4B	erythrocyte membrane protein band 4.1 like 4B	-23	Cytoplasm
MCAM	melanoma cell adhesion molecule	-24	Plasma Membrane
ITGB2	integrin subunit beta 2	-24	Plasma Membrane
PRSS8	serine protease 8	-24	Extracellular Space
ENTREP1	endosomal transmembrane epsin interactor 1	-25	Cytoplasm
AFDN	afadin, adherens junction formation factor	-25	Nucleus
OOEP	oocyte expressed protein	-25	Cytoplasm

ABCA3	ATP binding cassette subfamily A member 3	-25	Cytoplasm
KRT7	keratin 7	-25	Cytoplasm
TMT1A	thiol methyltransferase 1A	-26	Cytoplasm
CXADR	CXADR Ig-like cell adhesion molecule	-26	Plasma Membrane
UNC13B	unc-13 homolog B	-26	Cytoplasm
SLC39A4	solute carrier family 39 member 4	-26	Plasma Membrane
RALGAPA2	Ral GTPase activating protein catalytic subunit alpha 2	-27	Cytoplasm
BEND7	BEN domain containing 7	-28	Cytoplasm
MPP7	MAGUK p55 scaffold protein 7	-29	Plasma Membrane
EXPH5	exophilin 5	-33	Cytoplasm
Sec14l4	SEC14-like lipid binding 4	-33	Other
SFTPD	surfactant protein D	-34	Extracellular Space
KRT19	keratin 19	-35	Cytoplasm
CCR1	C-C motif chemokine receptor 1	-36	Plasma Membrane
ITGA3	integrin subunit alpha 3	-36	Plasma Membrane
MAOB	monoamine oxidase B	-37	Cytoplasm
VEPH1	ventricular zone expressed PH domain containing 1	-37	Nucleus
SORL1	sortilin related receptor 1	-37	Cytoplasm
LMO7	LIM domain 7	-38	Cytoplasm
F11R	F11 receptor	-38	Plasma Membrane
MME	membrane metalloendopeptidase	-39	Plasma Membrane
Ighg	Immunoglobulin heavy chain (gamma polypeptide)	-39	Extracellular Space
MYO5C	myosin VC	-40	Cytoplasm
EPCAM	epithelial cell adhesion molecule	-42	Plasma Membrane
DPEP1	dipeptidase 1	-43	Cytoplasm
ARHGEF26	Rho guanine nucleotide exchange factor 26	-44	Plasma Membrane
ATP6V1C2	ATPase H+ transporting V1 subunit C2	-46	Cytoplasm
TSPAN8	tetraspanin 8	-49	Plasma Membrane
LRP2	LDL receptor related protein 2	-51	Plasma Membrane
ICAM1	intercellular adhesion molecule 1	-51	Plasma Membrane
DSP	desmoplakin	-52	Plasma Membrane
EMP2	epithelial membrane protein 2	-52	Plasma Membrane
NPNT	nephronectin	-53	Plasma Membrane
OGFRL1	opioid growth factor receptor like 1	-57	Other
DRAM1	DNA damage regulated autophagy modulator 1	-69	Cytoplasm
Defb4/Defb5	defensin beta 4	-75	Extracellular Space
KCNK1	potassium two pore domain channel subfamily K member 1	-90	Plasma Membrane
SCNN1A	sodium channel epithelial 1 subunit alpha	-95	Plasma Membrane
PLET1	placenta expressed transcript 1	-96	Plasma Membrane
SULT1A1	sulfotransferase family 1A member 1	-99	Cytoplasm
FGG	fibrinogen gamma chain	-114	Extracellular Space
Anxa8	annexin A8	-115	Cytoplasm
CDH1	cadherin 1	-118	Plasma Membrane
AQP5	aquaporin 5	-133	Plasma Membrane
PERP	p53 apoptosis effector related to PMP22	-155	Plasma Membrane
PON3	paraoxonase 3	-157	Extracellular Space
ALCAM	activated leukocyte cell adhesion molecule	-198	Plasma Membrane
NDNF	neuron derived neurotrophic factor	-229	Extracellular Space
SLC6A14	solute carrier family 6 member 14	-449	Plasma Membrane
SLC34A2	solute carrier family 34 member 2	-596	Plasma Membrane
TACSTD2	tumor associated calcium signal transducer 2	-692	Plasma Membrane
MAL2	mal, T cell differentiation protein 2	-759	Plasma Membrane
PIGR	polymeric immunoglobulin receptor	-919	Plasma Membrane
CLDN18	claudin 18	-1057	Plasma Membrane
Cxcl3	C-X-C motif chemokine ligand 3	-1740	Extracellular Space
SCGB1A1	secretoglobin family 1A member 1	-2893	Extracellular Space
Sftpa1	surfactant associated protein A1	-4511	Extracellular Space
SFTPC	surfactant protein C	-5227	Extracellular Space

Supplementary Table S3

Table S3. Canonical pathways uniquely dysregulated in MWCNT-Induced MM.

	MWCNT-7-MM	1.5 µm-DWCNT-ADC	MWCNT-7 -ADC	7 µm-DWCNT -ADC
**Pathways uniquely upregulated in MWCNT-7-MM**				
Extracellular matrix organization	4.6	-	-	-
Collagen biosynthesis and modifying enzymes	4.3	-	-	-
Collagen degradation	3.6	-	-	-
Collagen chain trimerization	3.4	-	-	-
Striated Muscle Contraction	4.6	-	-	-
Actin Cytoskeleton Signaling	3.1	-	-	-
Pulmonary Fibrosis Idiopathic Signaling Pathway	3.5	-	-	-
Calcium Signaling	3.0	-	-	-
ABRA Signaling Pathway	2.8	-	-	-
Role of Osteoclasts in Rheumatoid Arthritis Signaling Pathway	2.8	-	-	-
Degradation of the extracellular matrix	2.8	-	-	-
SNARE Signaling Pathway	2.7	-	-	-
Glycosaminoglycan metabolism	2.5	-	-	-
Opioid Signaling Pathway	2.5	-	-	-
GP6 Signaling Pathway	2.4	-	-	-
Syndecan interactions	2.3	-	-	-
nNOS Signaling in Skeletal Muscle Cells	2.3	-	-	-
Signaling by PDGF	2.3	-	-	-
Activation of NMDA receptors and postsynaptic events	2.2	-	-	-
Assembly and cell surface presentation of NMDA receptors	2.2	-	-	-
NCAM signaling for neurite out-growth	2.1	-	-	-
ILK Signaling	2.1	-	-	-

TR/RXR Activation	2.1	-	-	-
EPH-Ephrin signaling	2.0	-	-	-
PFKFB4 Signaling Pathway	2.0	-	-	-
Oxytocin Signaling Pathway	2.0	-	-	-
**Pathways uniquely downregulated in MWCNT-7-MM**				
Th1 Pathway	-2.6	-	-	-
Neutrophil degranulation	-3.3	-	-	-
Neutrophil Extracellular Trap Signaling Pathway	-4.0	-	-	-
Surfactant metabolism	-2.8	-	-	-
Aryl Hydrocarbon Receptor Signaling	-2.0	-	-	-
DAP12 interactions	-2.0	-	-	-
Formation of Fibrin Clot (Clotting Cascade)	-2.1	-	-	-
Class A/1 (Rhodopsin-like receptors)	-2.2	-	-	-
Role of Hypercytokinemia/hyperchemokinemia in the Pathogenesis of Influenza	-2.2	-	-	-
Specification of primordial germ cells	-2.2	-	-	-
Dilated Cardiomyopathy Signaling Pathway	-2.8	-	-	-

The values listed are z scores.
"- " indicates that the pathway was not significantly upregulated or downregulated in that tumor model.

Supplementary Table S4

Table S4. Comparison of canonical pathways enriched across MWCNT-induced ADCs.

	1.5 µm-DWCNT-ADC	MWCNT-7-ADC	7 µm-DWCNT-ADC
**Pathways commonly upregulated in MWCNT-7-ADC and 7 µm-DWCNT-ADC but not in 1.5 µm-DWCNT-ADC**			
PTEN Signaling	-	2.1	2.7
PPAR Signaling	-	2.6	2.1
Transport of inorganic cations/anions and amino acids/oligopeptides	-	2.1	2.3
**Pathways commonly downregulated in MWCNT-7-ADC and 7 µm-DWCNT-ADC but not in 1.5 µm-DWCNT-ADC**			
Pulmonary Healing Signaling Pathway	-	-2.2	-3.0
Oxytocin Signaling Pathway	-	-2.4	-2.0
Signaling by NOTCH2	-	-2.0	-2.0
α-Adrenergic Signaling	-	-2.0	-2.0
Pulmonary Fibrosis Idiopathic Signaling Pathway	-	-2.4	-2.7
Pulmonary Healing Signaling Pathway	-	-2.2	-3.0
Immunogenic Cell Death Signaling Pathway	-	-2.2	-2.0
Insulin Secretion Signaling Pathway	-	-2.3	-2.0
Role of Osteoclasts in Rheumatoid Arthritis Signaling Pathway	-	-2.5	-2.0
Hepatic Cholestasis	-	-2.2	-2.1
Apelin Endothelial Signaling Pathway	-	-2.1	-2.1
GNRH Signaling	-	-2.5	-2.1
14-3-3-mediated Signaling	-	-2.3	-2.1
Chronic Myeloid Leukemia Signaling	-	-2.0	-2.1
Factors Promoting Cardiogenesis in Vertebrates	-	-2.1	-2.1
Nitric Oxide Signaling in the Cardiovascular System	-	-2.3	-2.1
Effects of PIP2 hydrolysis	-	-2.0	-2.2
Signaling by SCF-KIT	-	-2.0	-2.2
Oxytocin in Spinal Neurons Signaling Pathway	-	-2.2	-2.2
Corticotropin Releasing Hormone Signaling	-	-2.1	-2.3
GP6 Signaling Pathway	-	-2.1	-2.3
Apelin Cardiomyocyte Signaling Pathway	-	-2.3	-2.3
Cachexia Signaling Pathway	-	-3.3	-2.4
VEGF Signaling	-	-2.0	-2.4
Ephrin Receptor Signaling	-	-2.0	-2.4
G alpha (z) signalling events	-	-2.2	-2.4
WNK Renal Signaling Pathway	-	-2.4	-2.4
NCAM signaling for neurite out-growth	-	-2.0	-2.6
Signaling by NOTCH1	-	-2.4	-2.6
HGF Signaling	-	-2.6	-2.6
Role of Tissue Factor in Cancer	-	-2.8	-2.7
Colorectal Cancer Metastasis Signaling	-	-3.2	-2.7
Sertoli Cell-Germ Cell Junction Signaling Pathway (Enhanced)	-	-3.3	-2.7
Estrogen Receptor Signaling	-	-3.4	-2.7
P2Y Purinergic Receptor Signaling Pathway	-	-3.2	-2.7
Dopamine-DARPP32 Feedback in cAMP Signaling	-	-3.2	-2.7
Platelet homeostasis	-	-2.4	-2.8
Class B/2 (Secretin family receptors)	-	-2.6	-2.8
Regulation of the Epithelial Mesenchymal Transition by Growth Factors Pathway	-	-2.3	-2.9
VEGF Family Ligand-Receptor Interactions	-	-2.1	-3.0
ABRA Signaling Pathway	-	-2.2	-3.0
IL-8 Signaling	-	-2.8	-3.2
Wound Healing Signaling Pathway	-	-3.3	-3.8
Hepatic Fibrosis Signaling Pathway	-	-3.5	-3.9
**Pathways Uniquely upregulated in 1.5 µm-DWCNT-ADC but not in MWCNT-7-ADC and 7 µm-DWCNT-ADC**			
Cellular Effects of Sildenafil (Viagra)	2.4	-	-
Regulation of Insulin-like Growth Factor (IGF) transport and uptake by IGFBPs	2.3	-	-
Assembly of collagen fibrils and other multimeric structures	2.1	-	-
Post-translational protein phosphorylation	2.1	-	-
Apelin Adipocyte Signaling Pathway	2.0	-	-
TGF-β Signaling	2.0	-	-

Glycolysis I	2.0	-	-
**Pathways Uniquely downregulated in 1.5 µm-DWCNT-ADC but not in MWCNT-7-ADC and 7 µm-DWCNT-ADC**			
PI3K Cascade	-2.0	-	-
Semaphorin interactions	-2.0	-	-
Sensory processing of sound by inner hair cells of the cochlea	-2.0	-	-
Sensory processing of sound by outer hair cells of the cochlea	-2.0	-	-
Glutamate binding, activation of AMPA receptors and synaptic plasticity	-2.0	-	-
Phase I - Functionalization of compounds	-2.6	-	-

The values listed are z scores.

"- " indicates that the pathway was not significantly upregulated or downregulated in that tumor model.
